# Gut microbes exacerbate systemic inflammation and behavior disorders in neurologic disease CADASIL

**DOI:** 10.1186/s40168-023-01638-3

**Published:** 2023-09-08

**Authors:** Sheng Liu, Xuejiao Men, Yang Guo, Wei Cai, Ruizhen Wu, Rongsui Gao, Weicong Zhong, Huating Guo, Hengfang Ruan, Shuli Chou, Junrui Mai, Suning Ping, Chao Jiang, Hongwei Zhou, Xiangyu Mou, Wenjing Zhao, Zhengqi Lu

**Affiliations:** 1 Shenzhen Key Laboratory for Systems Medicine in Inflammatory Diseases, School of Medicine, Shenzhen Campus of Sun Yat-Sen University, Shenzhen, 518107 Guangdong China; 2 Department of Neurology, Center for the Study of Mental and Neurological Disorders, the Third Affiliated Hospital of Sun Yat-Sen University, Sun Yat-Sen University, Guangzhou, 510630 Guangdong China; 3 Life Sciences Institute, Zhejiang University, Hangzhou, 310012 Zhejiang China; 4 Department of Laboratory Medicine, Microbiome Medicine Center, Zhujiang Hospital, Southern Medical University, Guangzhou, 510280 Guangdong China

**Keywords:** CADASIL, Gut-brain axis, Microbiome, Inflammatory cytokines, *Fusobacterium varium*

## Abstract

**Background:**

Cerebral autosomal dominant arteriopathy with subcortical infarcts and leukoencephalopathy (CADASIL) is a cerebral small vessel disease that carries mutations in *NOTCH3*. The clinical manifestations are influenced by genetic and environmental factors that may include gut microbiome.

**Results:**

We investigated the fecal metagenome, fecal metabolome, serum metabolome, neurotransmitters, and cytokines in a cohort of 24 CADASIL patients with 28 healthy household controls. The integrated-omics study showed CADASIL patients harbored an altered microbiota composition and functions. The abundance of bacterial coenzyme A, thiamin, and flavin-synthesizing pathways was depleted in patients. Neurotransmitter balance, represented by the glutamate/GABA (4-aminobutanoate) ratio, was disrupted in patients, which was consistent with the increased abundance of two major GABA-consuming bacteria, *Megasphaera elsdenii* and *Eubacterium siraeum*. Essential inflammatory cytokines were significantly elevated in patients, accompanied by an increased abundance of bacterial virulence gene homologs. The abundance of patient-enriched *Fusobacterium varium* positively correlated with the levels of IL-1β and IL-6. Random forest classification based on gut microbial species, serum cytokines, and neurotransmitters showed high predictivity for CADASIL with AUC = 0.89. Targeted culturomics and mechanisms study further showed that patient-derived *F. varium* infection caused systemic inflammation and behavior disorder in *Notch3*^R170C/+^ mice potentially via induction of caspase-8-dependent noncanonical inflammasome activation in macrophages.

**Conclusion:**

These findings suggested the potential linkage among the brain-gut-microbe axis in CADASIL.

Video Abstract

**Supplementary Information:**

The online version contains supplementary material available at 10.1186/s40168-023-01638-3.

## Introduction

Cerebral autosomal-dominant arteriopathy with subcortical infarcts and leukoencephalopathy (CADASIL) is the most common form of hereditary cerebral small vessel disease (CSVD) in adults bearing with *NOTCH3* mutation [1, 2]. Predominant clinical features include migraines with aura, recurrent transient ischemic attacks or ischemic strokes, mood disturbances, cognitive disorder, and vascular dementia [3–5]. A recent study has revealed that the prevalence of pathogenic mutations of *NOTCH3* for CADASIL is 3.4/1000 worldwide, which is 100 times higher than previously estimated [6]. The high expense of diagnostics may even lead to an underestimation of the prevalence. There are currently no effective treatments that can prevent the progression of CADASIL, and patients who missed early diagnosis suffer from severe symptoms [7]. Although antiplatelet or thrombolysis therapies are commonly used in clinics to alleviate the symptoms such as migraines and strokes [8], the safety remains to be evaluated and clarified due to the risk of intracerebral hemorrhages in a considerable percentage of CADASIL patients. Considering the increased number and hyper-activation of perivascular macrophages [9], immuneregulatory treatment has been regarded as a promising therapeutic strategy. However, no effective immunotherapy of CADASIL has been proposed. In an attempt to develop curative treatment, a profound understanding of the pathogenesis of CADASIL is a must for the discovery of novel therapeutic targets and the development of effective treatment.

People carrying *NOTCH3* mutation exhibit an extremely broad range of severity from none to full symptoms, and one of the contributing factors is the different variants of *NOTCH3* mutations [10]. In addition to the genetic polymorphism, increasing evidence has highlighted the environmental effects on the progression of CADASIL. CADASIL patients carrying the same *NOTCH3* variant have suffered with different onset ages and severity of clinical manifestations [11, 12], even in monozygotic twins [13]. Moreover, CADASIL patients influenced by environmental factors such as smoking and alcohol consumption were more prone to stroke, a major symptom of CADASIL [14, 15]. Likewise, environmental factors have been involved in the pathogenesis of amyotrophic lateral sclerosis (ALS) [16, 17]. The onset and severity of Huntington’s disease (HD), another inherited autosomal-dominant neurodegenerative disorder [18], have also been reported under the influence of various environmental factors [19].

The gut microbiota has been recognized as the second genome of human and one of the most important environmental factors which participate in the pathogenesis of various diseases, including cerebrovascular diseases, psychiatric disorders, and neurological disorders [20]. Alterations in gut microbiota composition have been demonstrated to be associated with ALS [21, 22], autistic spectrum disorder (ASD) [23, 24], depression [25, 26], schizophrenia [27, 28], Parkinson’s disease [29, 30], Alzheimer’s disease [31], and HD [32]. Gut microbiota potentially exerts effects through the brain-gut-microbe axis and has been shown to modulate motor-neuron function in an ALS-prone *Sod1* transgenic mouse model [22]. Supplementation of *Akkermansia muciniphila* and its derived nicotinamide ameliorated the symptoms of ALS, providing an opportunity to identify modifiable environmental and microbial therapeutic targets [22]. Additionally, in an ASD mouse model (*Cntnap2*^−/−^), the gut microbiota has been shown to play roles in modulating social-behavior phenotype [33]. For CADASIL, a cohort study of 15 patients and 16 family-member controls has been conducted to explore the taxonomic change in gut microbiome, based on 16S deep sequencing of fecal samples [34]. It has been found that several genera were associated with the disease [34]. However, the roles and function of gut microbiota in CADASIL remain largely unknown.

To further investigate the brain-gut-microbe axis and biological mechanisms in CADASIL, we first conducted an integrated-omics analysis on 24 CADASIL patients and 28 healthy family members (study design shown in Fig. S1). We have obtained metagenomic data from fecal samples, untargeted metabolome data from fecal and serum samples, targeted neurotransmitter profiles from serum samples, ELISA-based cytokine profiles from serum samples, and cognitive evaluation. This integrated omics study demonstrated a tight linkage of the brain-gut-microbe axis in CADASIL patients. Furthermore, targeted culturing and mechanistic study demonstrated that patient-derived *F. varium* provoked caspase-8-dependent noncanonical inflammasome activation and subsequent pyroptosis in macrophages, which potentially contributed to the systemic inflammatory status and behavior disorder in *NOTCH3* mutant mice. Our study revealed the associations between several bacterial factors and CADASIL, and furthermore provided mechanistic insights on how *F. varium* may have contributed or involved with the inflammatory and behavior phenotypes of the disease.

## Methods

### Participants of the study population

Twenty-four CADASIL patients were recruited in this study. Individuals were diagnosed with CADASIL by medical doctors at the Third Affiliated Hospital of Sun Yat-sen University according to a combination of symptoms and *NOTCH3* genetic testing. In order to best control the genetic background and other factors that influence gut microbiota, we recruited 28 healthy household members that were age and sex matched to the 24 CADASIL patients. For each CADASIL patient, his/her spouse at a similar age was invited to join the cohort. If the spouse did not join the cohort, one of his/her siblings at a similar age was invited. All subjects were excluded if they had infectious diseases, neoplastic disease, autoimmune disorders, and diabetes mellitus or were administered with antibiotics, antifungals, or chronic immunosuppressive medication in the 3 months before participation. Clinical information was collected according to standard procedures, including age, gender, body mass index (BMI), self-rating anxiety scale, self-rating depression scale, and cognitive scores on the Montreal Cognitive Assessment (MoCA) [35]. This study complied with all relevant ethical regulations and was approved by the ethics committee of the Third Affiliated Hospital of Sun Yat-sen University with the ethical number 202002–148-01. Written informed consent was obtained from all subjects.

### Sample collection

For stool samples, seven days before fecal sample collection, subjects were not allowed to take any food containing probiotics such as yogurt. Stool samples from 52 subjects were collected by the clinicians using the Longseegen stool storage tubes (Guangdong Longsee Biomedical Co., Ltd., Guangzhou, China; no. LS-R-P-007) that contain 2-mL stabilization buffer and were transported to the laboratory and frozen at − 80 °C within 3 h until DNA extraction and metagenomic sequencing or untargeted metabolomics. For serum samples, peripheral venous blood (5 mL) was drawn after overnight fasting. Then, serum samples were obtained by centrifugation at 3000 r/min for 10 min. Subsequently, serum samples were stored at − 80 °C until untargeted LC–MS analysis, targeted neurotransmitters profiling, and ELISA cytokines assay.

### DNA extraction and metagenome sequencing

Fecal genomic DNA was extracted with the QIAamp Fast DNA stool mini kit (Qiagen). DNA concentration was measured using NanoDrop 2000 spectrophotometer (Thermo Scientific). For shotgun sequencing, Illumina libraries were prepared using a Nextera DNA Sample Prep kit (Illumina) according to the manufacturer’s protocol and sequenced using the Illumina NovaSeq platform with 2 × 150-bp paired-end reads.

### Metagenomic analysis

Illumina adapters and low-quality reads were filtered and trimmed by fastp version 0.20.0 with the default parameters. Human reads were removed by mapping the reads to the human reference genome (hg38) with Bowtie2 version 2.4.1 [36]. The composition of microbial communities was computed using MetaPhlAn version 3.0.13 with default parameters [37]. Gene families were annotated using UniRef90 identifiers, and the MetaCyc pathways were conducted by HUMAnN version v3.0.0 [38]. Then, they were quantile normalized and scaled. Biological classification and metabolic enzymes of the pathways were retrieved from the MetaCyc website (https://metacyc.org/). UniRef90 abundance profiles were converted to enzyme commission (EC) abundance profiles using the “uniref90_level4ec” option. α-Diversity was estimated based on the species profile of each sample according to the Shannon index. Principal coordinate analysis (PCoA) was performed to visually evaluate the difference and similarity of bacterial communities between groups (beta-diversity). The “ggcorrplot” package was used to visualize the correlation matrix of differential species. Species co-occurrence networks corresponding to the CADASIL patients and healthy controls were performed based on the Spearman correlation algorithms.

### The abundance of virulence factors (VFs) and source analysis

Analysis of bacterial virulence in the metagenome sequence data was performed using the virulence factor database (VFDB) [39] by Diamond v2.0.2.140 [40]. A total of 27,285 protein sequences of the full virulence factor dataset were downloaded from VFDB (version March 4, 2022, http://www.mgc.ac.cn/VFs/). We used the alignment option “-k 1” to keep the best match, and alignment was only considered valid if both paired reads aligned to the same protein sequence [41]. VFs protein abundances were quantified as counts per million (CPM): calculated by the number of valid alignments, divided by the library sizes, and multiplied by one million as previously described [41]. VFs proteins present in less than 5% of the samples were discarded, and finally, the abundances of 3110 VFs proteins were analyzed. Next, to figure out which species these virulence factors come from, we used the species genome from ChocoPhlAn pangenome database v296_v201901b to analyze their VFs with Diamond and VFDB.

### Untargeted metabolomics analysis

For fecal metabolomics, 44 samples (case, *n* = 20; control, *n* = 24) were completely homogenized with fivefold volume of 0 °C distilled water. After high-speed centrifugation (16,200 g for 15 min at 4 °C), water extractions were transferred to a new 2-mL tube. Meanwhile, quality control (QC) samples pooling all samples were individually prepared using the same protocol. For serum metabolomics, 22 serum (case, *n* = 12; control, *n* = 10) samples were thawed on ice, and QC sample was also made by mixing and blending equal volumes (10 μl) of each serum sample. Liquid chromatography-tandem mass spectrometer (LC–MS/MS) analyses were performed using a UHPLC system (Thermo Fisher Scientific, USA) with a UPLC BEH C18 column (1.7 μm, 2.1 mm × 100 mm, Waters, USA) coupled to a Q Exactive HF-X mass spectrometer (Thermo Fisher Scientific, USA) in both positive and negative ionization modes. Data pre-processing and statistical analysis were performed using the metabolomics R package metaX [42]. Metabolites and functions were identified using mzCloud (https://www.mzcloud.org/), HMDB database (http://www.hmdb.ca), and KEGG database (http://www.genome.jp/kegg/) as previously described [43]. Partial least squares discriminant analysis (PLS-DA) was applied to discriminate CADASIL individuals from healthy controls. The variable importance in projection (VIP) > 1.0, two-tailed Student’s *t*-test (*P* < 0.05), and fold change (< 0.8 or > 1.2) were used to analyze metabolites that differed between the patients and controls. Metabolic pathway enrichment analysis of differential metabolites was performed based on the KEGG database.

### Targeted neurotransmitters profiling

A panel of 39 neurotransmitters including dopamine, serotonin, glutamic acid, L-glutamine, and γ-aminobutyric acid (GABA) in our 22 serum samples (case, *n* = 12; control, *n* = 10) were quantitatively detected by external calibration curves for each metabolite using AB Sciex 6500 Triple Quad LC–MS/MS at Beijing Genomics Institution (Shenzhen, China). Protein precipitation was first performed on the serum samples, aliquots of 50 μL were drawn, and four volumes of methanol were added. After thorough vortex and centrifugation, an aliquot of 180-μL supernatant was pipetted for drying. The dried residues were stored at − 80 °C until analysis. Quality control (QC) samples were performed using the same method. The chromatographic column is Waters BEH C18 column (1.7 μm, 2.1 mm × 100 mm, Waters, USA). The mobile phase consisting of 0.1% formic acid in water (A) and methanol (B) was delivered at a flow rate of 0.35 mL min^−1^ and column temperature of 55 °C. The linear gradient elution program was as follows: 0–2 min, 2% B and 2.5–15 min, 20–80% B. Multiple reaction monitoring (MRM) was used in monitoring the results of all compounds. Differential neurotransmitters were screened as follows: *VIP* > 1.0, two-tailed Student’s *t*-test (*P* < 0.05), and ratio (< 0.8 or > 1.2).

### Serum cytokine measurements

The levels of six human serum cytokines IL-1β, IL-6, IFN-γ, TNF-α, C–C motif chemokine ligand 2 (CCL2), and CXC chemokine ligand 16 (CXCL16) were determined in 22 serum samples (case, *n* = 12; control, *n* = 10) using corresponding ELISA kits (Ruixin Biotech, Quanzhou, China; Website: ruixinbio.com). The catalog numbers are RX106152H, RX106126H, RX106205H, RX104793H, RX104010H, and RX106378H, respectively.

### Statistics

In the microbial composition analysis, permutational multivariate analysis of variance (PERMANOVA) analysis was performed using the “adonis” function in the “vegan” package. Differential abundance of species, functional pathways, level-4 EC, and virulence factors was tested by a two-tailed Wilcoxon rank-sum test in controls and CADASIL individuals. We analyzed Spearman’s association between differential VFs and serum cytokines and between species and serum cytokines. The analyses and visualizations were implemented in R software (v4.1.0). Other statistical analysis including Student’s *t*-test and one-way ANOVA with Dunnett’s test was also used in some experiments that are noted in the figure legends.

### Random forest classifier for CADASIL

We constructed a classifier to discriminate samples of healthy controls and CADASIL patients based on a random forest model with the gut metagenomic species and together with serum cytokine and neurotransmitters. These confounding factors were normalized by min–max normalization using the following formula: normalized = (x-min(x))/(max(x)-min(x)). Five trials of a tenfold cross-validation approach were applied to evaluate the performance of the prediction model. A total of 90% of the samples were used to train the classifier, and the remaining 10% samples were tested to obtain the AUC. All variables were ranked based on their variable importance and added sequentially into the model. The averaged cross-validation errors were plotted as the number of variables increased. The mean decrease accuracy (MDA) score, which reflects the importance of variables in the model, was given to each variable based on the increase in error caused by removing that variable from the predictors. The receiver operating characteristic curves (ROC) for the cohort were plotted, and the best predictive variables were identified based on the maximum area under the curve (AUC) using the pROC package in R software.

### Targeted culturomics for outstanding species in patient or control feces

Fresh stool samples from CADASIL patients or healthy controls were collected into a 50-mL centrifuge tube containing 5-mL sterile pre-reduced PBS [44, 45]. The tubes were then put into an air-tight jar containing an AnaeroPack oxygen absorber-CO_2_ generator (Mitsubishi Gas Chemical, #C-1) and transported into an anaerobic chamber (Shellab BACTRON EZ-2) within 1 h. Each sample was diluted from 10^1^ to 10^11^ folds in pre-reduced PBS, after vortexing resuspension, and then was plated immediately on Colombian sheep blood agar, YCFA agar, BHI agar, GAM agar, and FS agar, incubated under anaerobic conditions until colonies were observed. Colonies were picked based on phenotypic diversity and experience. Using the 27F and 1492R primer pair, by colony PCR of the full 16S rRNA gene and subsequent Sanger sequencing, then forward and reverse reads were combined and then classified using BLAST. *Fusobacterium varium*, *Megasphaera elsdenii*, and *Faecalibacterium prausnitzii* have been successfully isolated by FS agar plate, BHI medium, and GAM medium, respectively, and then, they were purified, and the whole genome was sequenced.

### Bone marrow-derived macrophage (BMDM) cultures and bacteria treatment

Bone marrow cells were isolated from the femurs and tibia of healthy C57/Bl6 wild-type donors (age = 8–12 weeks). Macrophage precursors were cultivated and differentiated into macrophages for 6 days in MCSF-conditioned RPMI1640 medium containing 10% FBS and 1% penicillin–streptomycin (PS). Inside an anaerobic chamber, bacterial suspension used in co-culture with BMDM is prepared by scraping the bacterial lawn from a solid plate into RPMI-1640 supplemented with 10% fetal bovine serum (FBS) and diluted with RPMI160 (containing 10% FBS) to a suitable concentration. The tube containing the bacterial suspension is sealed to prevent air infiltration before being transported out from the anaerobic chamber and into a cell culture hood. BMDM were treated with *F. varium* or *F. prausnitzii* (BMDM: bacteria = 1: 20) for 1 h.

### Flow cytometric analysis

BMDM were collected, washed with PBS, and subjected to fixation and permeabilization (Invitrogen, Intracellular Fixation & Permeabilization Buffer Set, 88–8824-00) and then stained with intracellular antibodies. The following antibodies were used: anti-IL6-FITC (eBioscience 11–7061-82, clone: MP5-20F3, 1:200), anti-TNFα-APC (eBioscience 17–7321-82, clone: MP6-XT22, 1:200), and IL-1β (Affinity, AF5103, polyclonal, 1:400). Secondary antibodies of anti-rabbit Alexa Fluor 405 (Invitrogen, 1:1000) were then used to conjugate the primary antibodies. Cell viability of BMDM was assessed with PI staining. For CASP8 expression analysis in circulating monocytes, peripheral blood was extracted, and red blood cells were removed with ACK lysis buffer. Monocytes were identified as CD45^+^CD11b^+^F4/80^+^ cells. The mean fluorescent intensity (MFI) of CASP8 was calculated.

### Immunofluorescence staining

BMDM were seeded on coverslips coated with poly-L-lysine (Sigma). After treatment, cells were fixed with 4% paraformaldehyde. Fixed BMDM were washed and incubated with primary antibodies overnight in PBS containing 0.03% Triton-X100 and 3% BSA. After washing, sections or cells were incubated with secondary antibodies for 1 h at room temperature. The following primary antibodies were used: rabbit anti-IL-1β (Affinity, AF5103, polyclonal, 1:400), rabbit anti-phospho-P65 (pP65) (CST, 3033, clone: 93H1, 1:500), rabbit anti-NLRP3 (CST, 15,101, clone: D4D8T, 1:400), rabbit anti-Caspase 8 (CASP8) (Affinity, AF6442, polyclonal, 1:400), and rabbit anti-GSDMD (Affinity, AF4012, polyclonal, 1:400). The following secondary antibodies were applied: anti-rabbit secondary antibody conjugated with Cy3 (Jackson ImmunoResearch Laboratories, 1:1000) and anti-rabbit secondary antibody conjugated with AF488 (Jackson ImmunoResearch Laboratories, 1:1000). DAPI Fluoromount-G (Southern Biotech) was applied to locate nucleus when indicated. When co-labeling rabbit anti-NLRP3 and rabbit anti-CASP8, a TSA multispectral immunofluorescence staining kit (Panovue 10,001,100,100) was applied. Phalloidin (Invitrogen A12379, 1:3000) was used to label actin. Propidium iodide (PI) was applied to identify dead cells.

### Western blot

Protein was extracted with RIPA lysis buffer (Sigma) from the ipsilateral brain or BMDM. A total amount of 40-μg protein of each sample was applied to Western blot experiments. Western blot was performed with the standard SDS-polyacryamide gel electrophoresis method and enhanced chemiluminescence detection reagents (Invitrogen, WP20005). The following primary antibodies were used: rabbit anti-NLRP3 (CST, 15,101, clone: D4D8T, 1:1000), rabbit anti-IL-1β (Affinity, AF5103, polyclonal, 1:1000), rabbit anti-GSDMD (Affinity, AF4012, polyclonal, 1:1000), mouse anti-GAPDH (Proteintech, 60,004–1-Ig, clone: 1E6D9, 1:3000), mouse anti-Caspase 1 (CASP8) (Santa Cruz Biotechnology, sc392736, clone: D-3, 1:500), rabbit anti-Caspase 8 (CASP8) (Affinity, AF6442, polyclonal, 1:400), rat anti-Caspase 11 (CASP11) (CST, 14,340, clone: 17D9, 1:1000), and mouse anti-BACT (Proteintech, 66,009–1-Ig, clone: 2D4H5, 1:3000). Immunoreactivity was assessed with ImageJ (NIH).

### CADASIL mouse model and breeding

*Notch3*^R170C/+^ mutant C57BL/6 J mice were designed and generated by Shanghai Model Organisms Center (China) using the Cas9-targeted single guide RNA of 5′TCTGGTACAACTTGCCGTCATGG 3′. The obtained F0 mice were characterized by PCR and sequencing using primer pairs: F-5′GCACCGCCCGATTCTCCT 3′ and R-5′TTCCCTGGGCACAACTTACTTACC 3′. F1 (*Notch3*^R170C/+^, heterozygous) mice were generated by breeding F0 and wild-type (WT) C57BL/6 J mice and were genotyped by sequencing *Notch3*. Then, F2 (*Notch3*^R170C/R170C^, homozygous) mice were generated by breeding F1 mice. In vitro fertilization (IVF) was used to generate more embryos and then implanted in the uterus to obtain offspring of *Notch3*^R170C/+^ mice and WT littermates. All mice used in this study were kept under specific pathogen-free conditions.

### Animal ethics statements

All animal experiments were performed in accordance with the National Institute of Health Guide for the Care and Use of Laboratory Animals. All procedures were approved by the Animal Ethics Committee at the School of Medicine, Sun Yat-sen University, with the ethical number SYSU-IACUC-MED-2022-B086.

### *Fusobacteriumvarium* administration

*F. varium* was grown on FS medium in an anaerobic chamber at 37 °C. For gavage, the bacteria were centrifuged in an anaerobic tube and suspended in anaerobic PBS to a concentration of  10^9^ colony-forming units (CFU)/mL. The *F. varium* suspension was removed from the anaerobic tube and immediately administered to the 4-week-old WT littermates and *Notch3*^R170C/+^ mice orally by gavage (0.1 ml) every 2 days for 5 consecutive weeks. The abundance of *F. varium* in feces or mucus of the colon was identified using a qPCR assay with specific primers and normalized to pan-bacterial primers targeting the 16S rRNA gene (UNI 16S). The bacterial primer sequences are listed in Table S1. After 4 weeks of administration, blood samples were collected after one day of rest, and feces samples were collected after another two days, while behavioral test was conducted after further 2 days.

### Open field test

Mice were gently and softly placed in the center of a black plastic open-field arena with white background (625 mm × 740 mm × 510 mm) and allowed to explore freely for 5 min. A video camera positioned directly above the arena was used to track the movement of each animal and recorded on a computer with TM-Vison software (Techman Instrument, Chengdu) to track the total distance and the amount of time spent in the center of the chamber compared to the edges. The open-field test is commonly used for measuring the exploratory behavior and general activity of animals. The test equipment was separated in a dark, sound-insulated, and closed environment; tracking instrument recognized the mouse central body point with infrared lasers and sensors. More time spent in the edges of the arena with less time spent in the center of the arena is interpreted as anxiety-like behavior. Before the test, the mice were acclimatized to the room for 1 h, and the arena was cleaned with 75% EtOH after every trial.

### Serum cytokine IL-1β measurements

Serum from WT and *Notch3*^R170C/+^ mice in the two treatments (PBS and *F. varium* gavage, *n* = 6 for each group) were collected. The levels of cytokine IL-1β were determined in 24 serum samples using ELISA kits (Ruixin Biotech, Quanzhou, China; #RX203063M) according to the manufacturer’s instructions.

## Results

### Clinical characteristics of the cohort

A total of 24 patients with CADASIL and 28 healthy family members were recruited for this study (Table 1). There were no significant differences in age, gender, or body mass index between the two groups. All patients in CADASIL were observed with various symptoms. Fourteen patients (58.3%) experienced anxiety, 8 patients (33.3%) showed depressive symptoms, and 10 patients (41.67%) have an ischemic stroke history, and the percentage of the patient group carrying the symptoms above was significantly higher compared to the control group (Table 1). MoCA (Montreal Cognitive Assessment) [35] is a brief cognitive evaluation method with high sensitivity for detecting cognitive impairment, and a score of 26 or higher is considered to be normal. The MoCA median of CADASIL is 24, which is significantly lower than that of their family members is 28 (*P* < 0.0001, Table S2), indicating the existence of cognitive impairment in the CADASIL group.

**Table 1:** Characteristics of the study population

	CADASIL patients (*N* = 24)	Healthy family members (*N* = 28)	*p*-value*
Age — median (range)	52 (32–68)	50 (31–65)	0.86
Female sex — counts (%)	11 (45.8%)	16(57.1%)	0.58
BMI — median (range)	21.0 (17.5–26.8)	21.9 (18.4–27.3)	0.56
Anxiety — counts (%)	14 (58.3%)	1 (3.6%)	< 0.0001
Depression — counts (%)	8 (33.3%)	0 (0%)	0.001
TIA or IS history — counts (%)	10 (41.67%)	0 (0%)	0.0001
MoCA — median (range)	24 (8–30)	28 (26–30)	< 0.0001

^*^For *p*-value calculation, the *t*-test (age, BMI, and MoCA) or Fisher’s exact test (sex, anxiety, depression, and ischemic stroke history) was employed

### Altered composition of gut microbiota in CADASIL patients based on metagenomic data

**Fig. 1:**
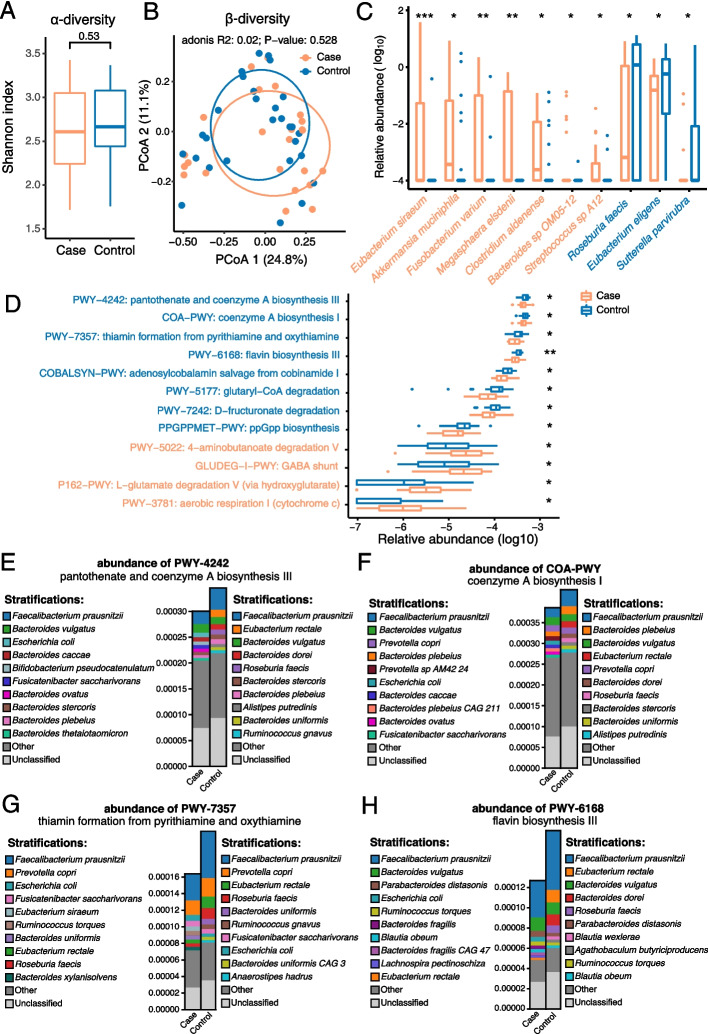
Fecal metagenomics analysis revealed the altered microbial and functional composition of gut microbiota in CADASIL patients. **A** The alpha diversity of the metagenomic samples. **B** PCoA of the gut microbial composition at species level based on the Bray–Curtis distance metric for patients and controls. Adonis, *R* = 0.02, *p* = 0.528. **C** Significantly different species in relative abundance between CADASIL patients and controls. Species names were colored according to the direction of enrichment, and orange and blue represent the species enriched in the case group and the control group, respectively. **D** The significantly different functional pathways in relative abundance between case and control groups. Pathway names were colored according to the direction of enrichment. The Wilcoxon rank-sum test was used to compare the abundance of species and pathways between the two groups. *p-value < 0.05; **p-value < 0.01; ***p-value < 0.001. **D**–**G** The top 4 differential pathways enriched in the control group including **E** PWY-4242 (pantothenate and coenzyme A biosynthesis III), **F** COA-PWY (coenzyme A biosynthesis I), **G** PWY-7357 (thiamin formation from pyrithiamine and oxythiamine), and **H** PWY-6168 flavin biosynthesis III (fungi) and the average relative abundance of their contributional species in two groups of samples, respectively. Species and “unclassified” stratifications are linearly (proportionally) scaled within the total bar height

The fecal samples from patients and controls were deep sequenced. After quality control and removing host sequences, 3,694,182,202 high-quality clean reads were obtained, with an average amount of 71,041,965 for each sample (Table S3). Within-sample alpha diversity analysis showed that the diversity index of microbial species was not significantly altered between patients and controls (Fig. 1A, Table S4). This is consistent with a previous report in which no significant difference in alpha diversity was observed between CADASIL patients and controls of a Japanese cohort via a 16S rDNA sequencing approach [34]. Furthermore, PCoA was performed to compare the extent of the similarity of the microbial communities in the two groups based on the Bray–Curtis distance (Fig. 1B). The result also indicated that the microbiota composition of the CADASIL patients was not significantly different from that of the family controls.

Next, we investigated whether there were specific genera and species differing in the microbial community composition between the two groups. The two-tailed Wilcoxon rank-sum test analysis showed the relative abundance of three genera was significantly altered. In particular, the patient group was characterized by a higher abundance of *Porphyromonas* and *Akkermansia*, while the control group harbored more abundant *Sutterella* in the gut (Fig. S2). At species level, seven species of *Eubacterium siraeum*, *Akkermansia muciniphila*, *Fusobacterium varium*, *Megasphaera elsdenii*, *Clostridium aldenense*, *Bacteroides* sp. OM05-12, and *Streptococcus* sp. A12 were significantly more abundant in patients, while a higher abundance of *Roseburia faecis*, *Eubacterium eligens*, and *Sutterella parvirubra* were present in the control group (Fig. 1C and Table S5). To assess the potential association of these species, the correlation coefficient of abundance between each of them was performed individually (Fig. S3). Interestingly, *F. varium* showed a significant positive correlation with *C. aldenense* in both patients (*R* = 0.58) and their family members (*R* = 0.9), which suggests possible symbiotic interactions between the two bacteria.

To describe potential relationships among major species in the gut bacterial community, we constructed co-occurrence networks of the top 30 abundant species based on significant Spearman correlations. As shown in supplemental Fig. S3, the CADASIL and control groups featured distinct patterns of correlation networks. The 30 major species of the control group exhibited a modest complexity of correlation, while the complexity in the CADASIL group was remarkably increased (Spearman’s correlation value <  − 0.4 or > 0.4). Taken together, our data revealed and characterized the gut microbiota alteration that occurs in CADASIL patients.

### Abnormal microbial functions of gut microbiota in CADASIL patients

To further evaluate the functions of gut microbiota, we performed functional analysis on fecal metagenomic data. Five-hundred MetaCyc pathways were obtained in 52 samples in our study. The abundance of 12 pathways was significantly altered, with 4 pathways enriched in the case group (Fig. 1D and supplemental Table S6), including 4-aminobutanoate (GABA) degradation V (PWY-5022), GABA shunt (GLUDEG-I-PWY), and L-glutamate degradation V (P162-PWY). Interestingly, these three pathways were involved with the metabolism of glutamate and GABA, which are excitatory and inhibitory neurotransmitters in the mammalian central nervous system, respectively. On the contrary, the pathways of coenzyme A biosynthesis (PWY-4242 and COA-PWY), thiamine formation (PWY-7357), and flavin biosynthesis III (PWY-6168) were depleted in the patient group (Fig. 1D). Coenzyme A, thiamin (vitamin B1), and flavin (vitamin B2) have been demonstrated essential for human health. Therefore, we decided to focus on the four related pathways (PWY-4242, COA-PWY, PWY-7357, and PWY-6168) for further investigation. We investigated the contributing bacterial species for these pathways, and the top 10 contributors to each pathway in each group were assessed (Fig. S4, Fig. 1E–H). Based on the observation, the list of the top 10 contributing bacteria in patients altered dramatically compared to that in controls.

We have plotted the abundance of each pathway from the top 5 contributing bacteria in controls (Fig. S5) and found *Roseburia faecis*, which made a substantial contribution for three pathways (PWY-4242, PWY-7357, and PWY-6168), was significantly decreased in patients (*p* < 0.05). Notably, the contribution from *Faecalibacterium prausnitzii* and *Eubacterium rectale* was also decreased in patients, although the change was not statistically significant. Similarly, we observed the abundance of *R. faecis* was significantly decreased in patients (also shown in Fig. 1C). We speculate the depletion of *R. faecis*, and the possible depletion of *F. prausnitzii* and *E. rectale* may cause the deficiency of biosynthesis pathways of coenzyme A, thiamine, and flavin, which hypothesis to be tested in the future. In summary, our results exhibited abnormal microbial function in CADASIL patients compared to controls.

### Aberrant metabolic configuration in CADASIL patients

Growing evidence showed some microbial-derived metabolites enter the bloodstream and exert their impacts on the physiology and behavior of the hosts [46]. To our knowledge, the metabolome of CADASIL patients has never been assessed. To fill in the blanks, we explored the metabolic profiles of fecal samples in CADASIL patients and their family members in the same household. In order to maximally bridge the potential linkage between gut microbiota-derived metabolites and systemic circulation, a serum metabolome analysis was also conducted.

**Fig. 2:**
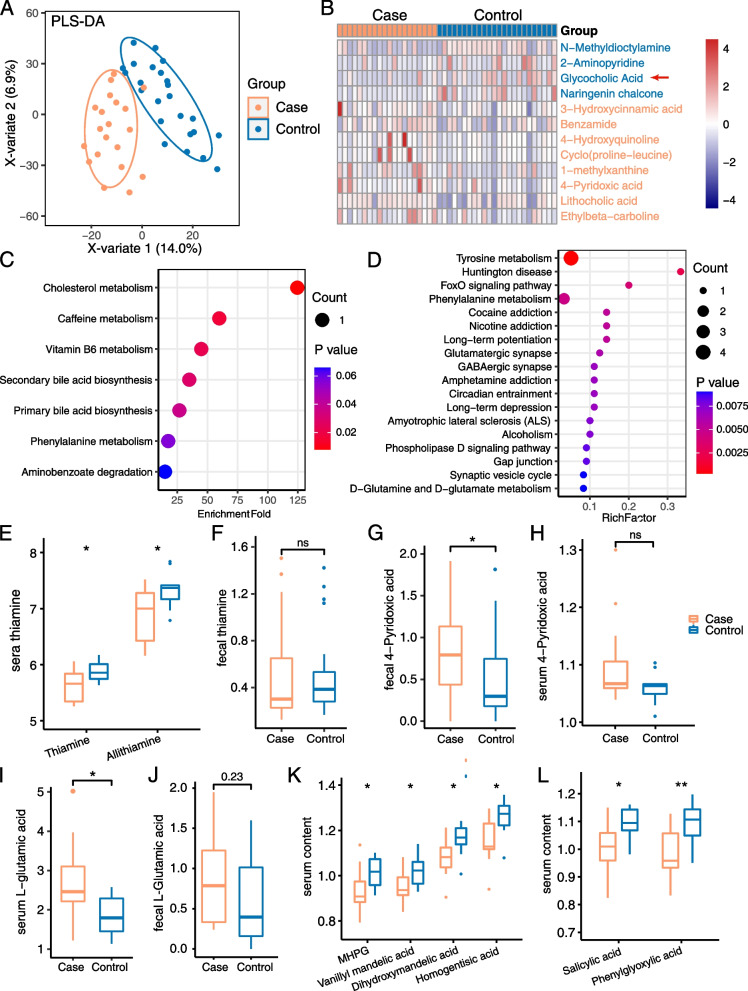
Fecal and serum metabolomics analysis revealed the metabolism activity was shifted in CADASIL patients compared to healthy controls. **A** The result of PLS-DA for fecal metabolome. Case, *n* = 20; control, *n* = 24. Samples in color orange are from the case group, while samples in blue are from the control group. **B** Heat map of the significantly altered fecal metabolites in case and control groups. Data are normalized based on the peak intensity. Metabolite names were colored according to the direction of enrichment. Metabolites in orange indicate they were upregulated in the case group. **C**–**D** Bubble plot of the notably enriched KEGG pathways according to differential fecal metabolites (**C**) and serum metabolites (**D**). The point size indicates the count of differential metabolites that involve in the corresponding pathways, and the color indicates the *p*-value. The rich factor is the ratio of differential metabolites number annotated in this pathway to all metabolites number annotated in this pathway. **E** The content of thiamine including common thiamine and allithiamine (a fat-soluble form of thiamine) in serum samples from CADASIL patients and healthy controls. **F** The content of thiamine in fecal samples from CADASIL patients and healthy controls. **G**–**H** The comparison of the content of 4-pyridoxic acid between the two groups of samples in feces (**G**) and serum (**H**). **I**–**J** The comparison of the content of L-glutamic acid between the two groups of samples in feces (**I**) and serum (**J**). **K** The serum content of four differential metabolites, related to tyrosine metabolism, between case and control groups. **L** The serum content of two differential metabolites, related to phenylalanine metabolism, between case and control groups. **E**–**L** The content of metabolites was normalized by the intensity of metabolite peaks, and the significance was tested by Student’s *t*-test. *p-value < 0.05; **p-value < 0.01; ns, not significant

PLS-DA was performed to visually evaluate the difference in fecal metabolites between the two groups (Fig. 2A). In fecal samples, eight known metabolites including 3-hydroxycinnamic acid, 4-pyridoxic acid, and lithocholic acid were upregulated in CADASIL patients, while four others such as glycocholic acid and N-methyldioctylamine were downregulated (Fig. 2B and Table S7), and KEGG pathway enrichment analysis indicated eight KEGG pathways were enriched in patients (Fig. 2C and Table S8). In serum, 23 known metabolites were upregulated in CADASIL patients, while 72 others were downregulated (Table S9), and KEGG pathway enrichment analysis indicated 32 KEGG pathways were enriched in patients (*P* < 0.05; Table S10). Furthermore, 18 KEGG pathways including tyrosine metabolism, Huntington disease, long-term potentiation, glutamatergic synapse, and GABAergic synapse were significantly enriched in patients with *p* < 0.01 (Fig. 2D).

In the previous section, we described bacterial biosynthesis pathways of vitamin B1, B2, and coenzyme A were diminished in CADASIL patients (Fig. 1D). Therefore, we next examined essential vitamins and coenzymes in both fecal and serum metabolome data. Notably, we detected that thiamine was significantly reduced (*p* = 0.018) in the serum of patients (Fig. 2E), which coincided with our observation of a depleted thiamine formation pathway in patients (Fig. 1D). Moreover, allithiamine, a fat-soluble form of thiamine that is more readily absorbed by the gastrointestinal tract [47], was also down-regulated in the serum of patients (Fig. 2E). The level of thiamine was also lower in the fecal samples of patients, although the difference was not statistically significant (Fig. 2F). We have also observed that 4-pyridoxic acid, a catabolic product of the B6 vitamins, was significantly increased in the feces of CADASIL patients (Fig. 2G). In serum, the level of 4-pyridoxic acid was also elevated, although the difference was not statistically significant (Fig. 2H).

Besides B vitamins, we observed the level of L-glutamic acid was significantly increased in the serum of patients. L-glutamic acid (Glu) is an essential excitatory neural transmitter that is related to multiple KEGG pathways including Huntington disease, long-term depression, and ALS, which were significantly enriched in the case group (Fig. 2I). In fecal samples, glutamate was also elevated although the change was not statistically significant (Fig. 2J). Taken together, our observation of the increased level of L-glutamic acid well coincided with the elevated levels of three related bacterial pathways (GABA degradation V, GABA shunt, and L-glutamate degradation V) in our previous analysis (Fig. 1C).

Another notably altered metabolite in feces was glycocholic acid (arrowed in Fig. 2B), which is an essential metabolite associated with more than one KEGG pathway, including cholesterol metabolism, secondary bile acid biosynthesis, and primary bile acid biosynthesis (Fig. 2B, Table S8); however, we did not observe an altered level of glycocholic acid in serum. Four significantly altered metabolites in serum (Fig. 2K, Table S9), dihydroxymandelic acid, vanillyl mandelic acid, MHPG (3-methoxy-4-hydroxyphenylethyleneglycol), and homogentisic acid, were included within the same KEGG pathway, tyrosine metabolism pathway. Two other altered metabolites (Fig. 2L), salicylic acid, and phenylglyoxylic acid were involved in the phenylalanine metabolism pathway. Tyrosine and phenylalanine metabolism were both significantly decreased in the serum of patients (Fig. 2D, Table S10). In short, our data indicated aberrant metabolic patterns in CADASIL patients.

### Disrupted Glu/GABA balance in CADASIL patients

Glutamate is closely related to 4-aminobutyric acid (GABA), which is an important inhibitory amino acid neurotransmitter in the mammalian central nervous system. Our functional analysis of fecal metagenomics has uncovered that three bacterial pathways of glutamate/GABA metabolism (Fig. 1C) were abnormal in patients, and the analysis of metabolome detected the increased level of L-glutamic acid in sera and feces (Fig. 2I, J). This coincidence led us to further assess the levels of GABA in addition to L-glutamate. Our result showed the fecal GABA was slightly decreased in patients but not statistically significant, while the serum GABA was roughly equal in patients compared to controls (Fig. S6).

**Fig. 3:**
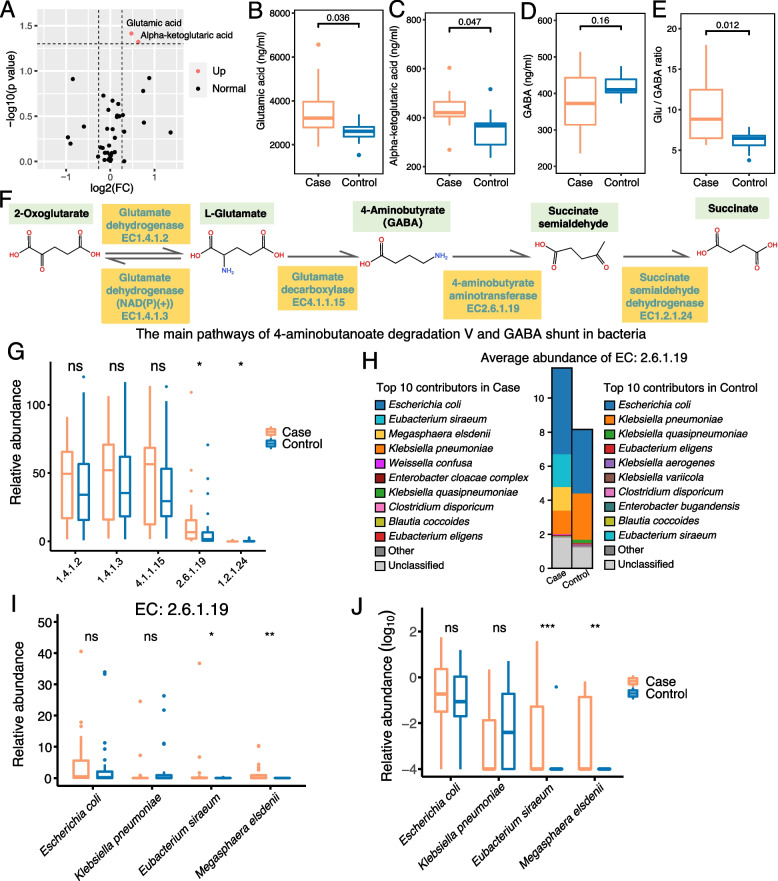
Glu level and GABA metabolism were significantly altered in CADASIL patients*.* **A** Volcano plot of the significantly different neurotransmitters between serum samples from the case and control groups. “Up” indicates the neurotransmitter was upregulated in the case group compared to the control group. **B**–**D** The content of serum glutamic acid (**B**), alpha-ketoglutaric acid (**C**), and GABA (**D**) in the samples from the case and control groups were identified by targeted neurotransmitter profiling. **E** The comparison of Glu/GABA ratio in serum samples between the case and the control group. **A**–**E** Case, *n* = 12; control, *n* = 10. **F** The schematic diagram of the main pathway about 4-aminobutanoate (GABA) degradation V and GABA shunt in bacteria and involved enzymes. **G** The abundance of the five enzymes in fecal metagenomic samples from patients and healthy controls. **H** Stacked bar plot of the average relative abundance of the top 10 contributing species to EC2.6.1.19 in the case and control groups, respectively. **I** The relative abundance of EC2.6.1.19 contributed by the top four species in the case and control groups. **J** The relative abundance of these four species in fecal metagenomics samples. **G**–**J** Case, *n* = 24; control, *n* = 28. Significance levels between the case and control groups were tested by Student’s *t*-test (**B**–**E**) and Wilcoxon rank-sum test (**G**, **I**, **J**). *p-value < 0.05; **p-value < 0.01; ns, not significant

In order to examine neurotransmitter profiles more accurately, we employed a targeted metabolomic approach on serum. Analysis of a panel of 39 neurotransmitter-related compounds revealed glutamic acid, and one of its ketone derivatives, alpha-ketoglutaric acid, were significantly increased (*p* < 0.05, *FC* > 1.3, and *VIP* > 1) in patients compared to controls (Fig. 3A–C, Table S11). The increased level of glutamic acid was consistent with our observations in the untargeted metabolome of serum (Fig. 2I). Glutamic acid (Glu) and GABA are excitatory/inhibitory amino acid neurotransmitters in the mammalian central nervous system, and the Glu/GABA ratio is a measurement of the excitatory/inhibitory balance [48]. Although the serum level of GABA was not significantly differential (Fig. 3D), the Glu/GABA ratio was elevated in patients significantly (Fig. 3E, Table S12; *p* = 0.012), suggesting a neurotransmitter dysregulation in CADASIL patients.

Gut microbiota has been reported to affect Glu/GABA ratio by the metabolism of Glu and GABA [48]. Next, we aimed to find out the attributing bacterial enzymes and species. First, we examined the pathways of 4-aminobutanoate degradation V and GABA shunt in bacteria that were related to Glu/GABA metabolism. The main enzymes participating in these reactions were summarized in Fig. 3F, which include glutamate dehydrogenase (EC1.4.1.2), glutamate dehydrogenase (NAD(P)( +)) (EC1.4.1.3), glutamate decarboxylase (GAD, EC4.1.1.15), 4-aminobutyrate aminotransferase (GABA-T, EC2.6.1.19), and succinate semialdehyde dehydrogenase (EC1.2.1.24). Furthermore, we found out that the gene abundance of EC2.6.1.19 and EC1.2.1.24 was elevated in patients (Fig. 3G).

In order to dissect the potential roles of specific bacterial species, we analyzed the top 10 attributing species for genes encoding EC2.6.1.19 (Fig. 3H, Fig. S7). Our results indicated that *Escherichia coli* and *Klebsiella pneumoniae* were the solely two species that make major contribution in the control group; however, there were four major contributing species in CADASIL patients including the additional species, *Eubacterium siraeum* and *Megasphaera elsdenii*. The abundance of genes encoding EC2.6.1.19 from *E. siraeum* and *M. elsdenii* was higher in patients (Fig. 3I), which coincided with the elevated abundance of the two bacteria in patients (Fig. 3J, also in Fig. 1B). Taken together, our results suggest the possibility that the increased levels of *E. siraeum* and *M. elsdenii* may exert as a cause for the elevated level of EC2.6.1.19 in patients.

### Elevated levels of inflammatory cytokines coincide with an enriched abundance of virulent gene homologs in the gut microbiota of CADASIL patients

**Fig. 4:**
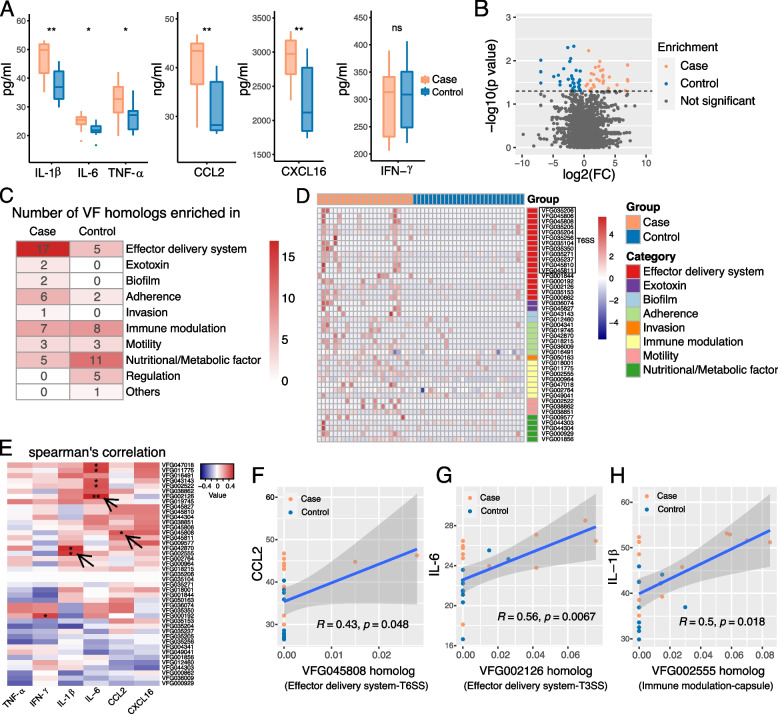
The level of serum inflammatory cytokines was elevated in CADASIL patients, and they were associated with virulence factors (VFs). **A** The level of six serum inflammatory cytokines was measured by ELISA in two groups of samples. Case, *n* = 12; control, *n* = 10. The significance levels were tested by Student’s *t*-test. *p-value < 0.05; **p-value < 0.01; ns, not significant. **B** Volcano plot of the significantly different VFs between metagenomic samples from case (*n* = 24) and control group (*n* = 28). Forty-three VFs were enriched in the case group (color in orange), while 35 VFs were enriched in the control group (color in blue). NS indicates not significant. *p*-value < 0.05 was set as the threshold. **C** Heat map of the number of significantly different VF homologs belonging to each category between the case and control groups. **D** Heat map of the abundance of 43 VFs that enriched in the case group. VFs belonging to the same category were listed together, and these VFs were divided into eight categories. **E** Heat map of the Spearman’s correlation between the enriched VFs in the case group and six serum inflammatory cytokines. Red indicates the positive correlation, while blue indicates the negative correlation. *p-value < 0.05; **p-value < 0.01. **F**–**H** Spearman correlation between the abundance of VFG045808 and serum level of CCL2 (**F**), between the abundance of VFG002126 and serum level of IL-6 (**G**), and between the abundance of VFG002555 and serum level of IL-1β (**H**) in 22 subjects (case, *n* = 12, color in orange; control, *n* = 10, color in blue). A total of 95% confidence interval around smooth was displayed, and Spearman rank correlation coefficient and *p*-values were shown in the plot

Previous research has suggested a possible link of inflammation in the pathology of CADASIL [49]. Next, we examined the systemic inflammation level of patients. The serum inflammatory cytokines were analyzed by ELISAs and revealed that the levels of serum IL-1β, IL-6, TNF-α, CCL2, and CXCL16 were significantly elevated in the patient group (Fig. 4A, Table S13) compared with their family members, suggesting a systemic inflammation in CADASIL patients.

Bacterial virulence factors have been demonstrated to induce local or systemic inflammation [50]. In the context of gut commensal bacteria, a virulence factor usually contributes to the pathogenic potential of commensal bacteria through several mechanisms, including increased adhesion of bacteria to the gut mucosa, evasion of the immune system, or suppression of the host immune response [41]. To evaluate the pathogenic and pro-inflammatory potentials of gut microbes, we assessed the homology between our metagenomic reads and the virulence factors from VFDB, a database for bacterial virulence genes [39]. Results showed there was no difference between the total abundance of virulence factor (VF) homologs between patients and controls (Fig. S8A); however, the abundance of 43 VF homologs was significantly increased (*p* < 0.05) in CADASIL patients, while only 35 were increased in controls (Fig. 4B, Table S14, and Table S15). In addition, more VF homologs from the categories effector delivery system, exotoxin, biofilm, adherence, and invasion were enriched in patients compared to controls (Fig. 4C). The heat map of VF homolog enriched in the case group was presented in Fig. 4D. In the effector delivery system category, 12 VF homologs related to bacterial type VI secretion system (T6SS, boxed in Fig. 4D) were enriched in patients (Fig. S8B), while only one (boxed in Fig. S9A) was enriched in control, suggesting T6SS gene homologs were enriched in CADASIL patients.

Besides the effector delivery system, two homologs to exotoxins (indicated by purple bars in Fig. 4D) and two homologs (indicated by light blue bars in Fig. 4D) related to biofilm formation were enriched in patients, while none homologs related to these two categories were enriched in controls. In addition, six homologs related to adherence (indicated by light green bars in Fig. 4D) were enriched in patients, while only two were enriched in controls. Taken together, our data demonstrated bacterial virulence factor genes related to T6SS, exotoxins, biofilm formation, and adherence were enriched in the gut microbiota of patients.

Furthermore, we examined the associations between the levels of each of the six tested cytokines and the abundance of each of the 43 VF homologs increased in patients as well as the 35 VF homologs in controls. We observed 9 positive correlated cytokine-VF pairs in patient-enriched VF homologs (Fig. 4E and Table S16) and none in control-enriched VF homologs (Fig. S9B). Interestingly, there were 23 negatively correlated cytokine-VF pairs in control-enriched VF homologs (Fig. S9B), suggesting VF homologs to VFDB are not necessarily inflammation promoting. Specifically, the level of CCL2 correlated with VFG045808, the tip protein of T6SS, and the top correlated VF homolog with IL-6 was VFG002126, the conserved export gate protein of T3SS apparatus (arrowed in Fig. 4E, Fig. 4F–G). These observations further suggest the possible roles of bacterial T6SS and T3SS in promoting inflammations in CADASIL patients. The highest correlated VF homolog with IL-1β was VFG002555, *Burkholderia pseudomallei* WcbL, a sugar kinase involved in the biosynthesis of capsular polysaccharides (arrowed in Fig. 4E, Fig. 4H). We compared VFG002555 with predicted proteins in our metagenomic data by BLASTp, and observed *Fusobacterium varium*, the patient-enriched species, possess proteins with the highest identities (> 50%) to VFG002555 (Table S17).

**Fig. 5:**
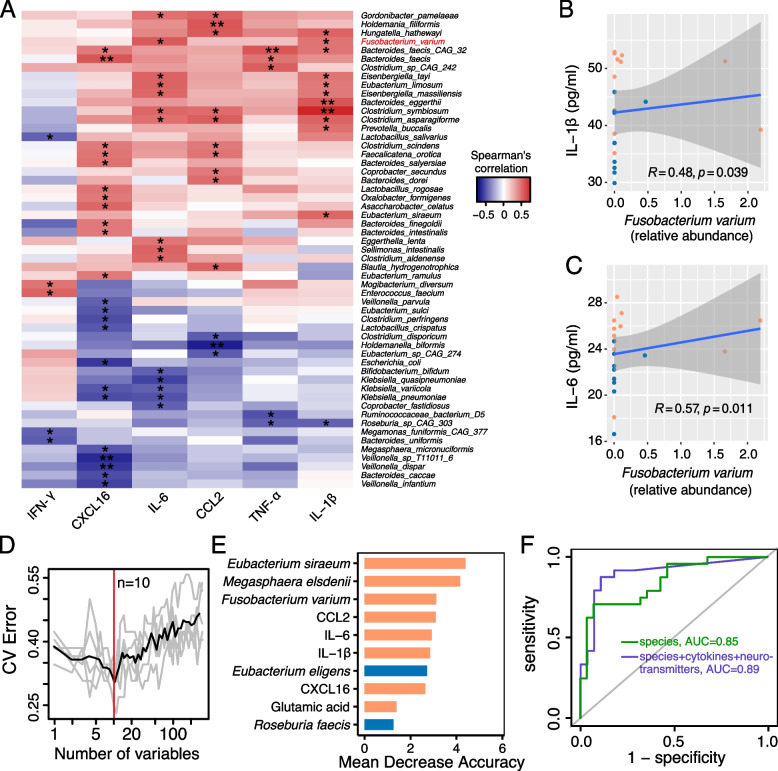
Association between the gut microbiota and serum inflammatory cytokines and the random forest classifier for CADASIL. **A** Heat map of the Spearman’s correlation between the abundance of species and six serum inflammatory cytokines. Red indicates the positive correlation, while blue indicates the negative correlation. *p-value < 0.05; **p-value < 0.01. Arrows point out the correlation of three species that were more abundant in the case group and corresponding inflammatory cytokines. **B**–**C** The Spearman’s correlation between the abundance of *Fusobacterium varium* and serum level of IL-1β (**B**) and IL-6 (**C**). Samples in orange and blue represent they are from the case group and the control group, respectively. Spearman’s rank correlation coefficient and *p*-values were shown. **D** The random forest model was trained using the abundance of fecal metagenomics species, the level of serum cytokines, and targeted neurotransmitters in our cohort. All variables were first ranked based on their variable importance and then added sequentially into the model. The error curves were plotted for the five trials of tenfold cross-validation in random forest classification as the number of variables increased. The black curve indicates the average cross-validation error of the five trials (in gray). The minimum error in the averaged curve plus the standard deviation at that point was used as the cutoff for feature selection. The model containing the smallest number of variables with an error below that cutoff was chosen as the optimal classifier. The red line marks the number of variables in the optimized model (*n* = 10). **E** The mean decrease accuracy (MDA) score of potential ten markers in the model. The colors represent enrichment or increase in CADASIL (orange shades). **F** Receiver operating characteristic (ROC) curve and the performance of the random forest model using the metagenomic species (green) and further a combination with serum cytokines and targeted neurotransmitters (blue)

We have also examined the associations between the levels of each cytokine and the abundance of each bacterial species. There were 47 positive related cytokine-bacteria pairs and 26 negative ones (Fig. 5A). Notably, patient-enriched *Fusobacterium varium* positively correlated with the level of IL-1β as well as IL-6 (Fig. 5B–C). Another patient-enriched bacterium, *Clostridium aldenense*, positively correlates with the level of IL-6. Our observations suggest the two bacteria may have inflammation-promoting potentials. To summarize, CADASIL patients exhibited elevated levels of inflammatory cytokines, along with the enrichment of specific bacteria and bacterial virulence gene homologs related to T6SS, T3SS, exotoxins, biofilm formation, and adherence, in their gut microbiota.

### Random forest analysis discovered potential biomarkers related to CADASIL

The distinct microbial differences, serum cytokines and neurotransmitters differences between CADASIL patients and healthy controls prompted us to investigate if they had the potential to discriminate CADASIL patients from healthy controls. A disease classifier was constructed based on the microbial species profile and serum profile using a random forest model in our cohort (Fig. 5D). After feature selection based on tenfold cross-validation (see “Methods”), ten features including  five bacterial species, four serum cytokines, and one serum neurotransmitter were retained with optimal performance (Fig. 5E). When using only bacterial species as biomarkers for CADASIL, the area under the receiver operating curve (AUC) is 0.85 (Fig. 5F). And when using all these features, the AUC increased to 0.89. This analysis demonstrated that ten features including *E. siraeum*,* M. elsdenii*, *F. varium*, CCL2, IL-6, IL-1β, *E. eligens*, CXCL16, glutamic acid, and *R. faecis* are potential biomarkers for diagnostics of CADASIL.

### Patient-derived *Fusobacteriumvarium* provokes noncanonical inflammasome activation in macrophages

Previous integrated analysis of our study has revealed several hypotheses that specific gut microbes might participate in the pathogenesis of CADASIL, which include the hypothesis that the patient-enriched *F. varium* and *Clostridium aldenense* may promote inflammation in CADASIL. Chronic inflammation is a key driving mechanism in a number of diseases. Therefore, we decided to prioritize our focus on *F. varium* and *C. aldenens*, especially the more abundant *F. varium*. To obtain *F. varium* culture, we carried out a targeted culturomics optimized for six bacteria including *F. varium*, *C. aldenense*, *E. siraeum*, *M. elsdenii*, *F. prausnitzii*, and *R. faecis*, with a focus on *F. varium*. From the feces of CADASIL patients and healthy controls, we recovered 193 species including *F. varium*, *M. elsdenii*, and *F. prausnitzii*.

**Fig. 6:**
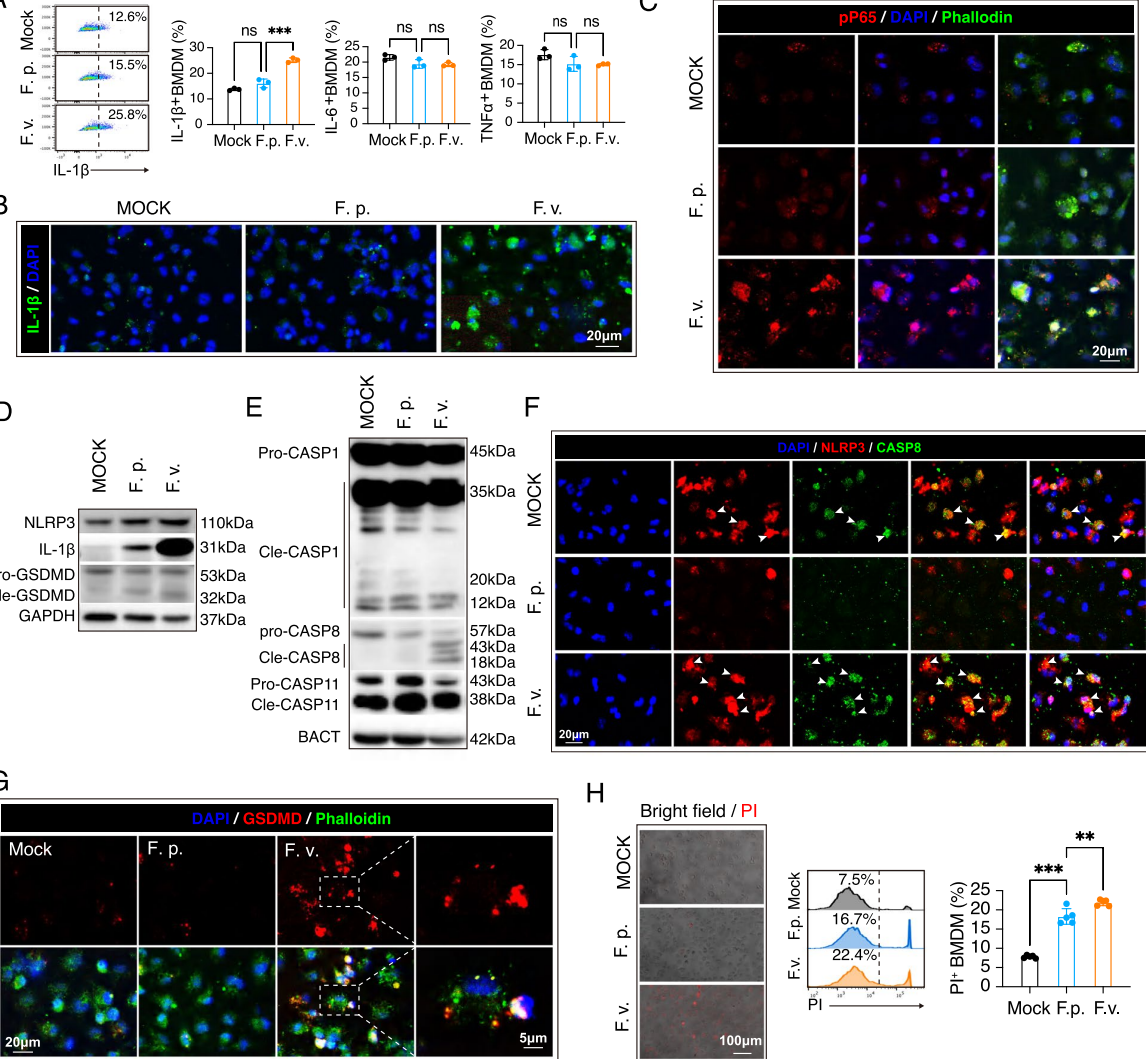
*Fusobacterium varium* induces noncanonical Caspase 8 inflammasome activation in macrophages. Bone marrow-derived macrophages (BMDM) from healthy wild-type C57/Bl6 mice (age = 8–12 weeks) were cultured and treated with *F. varium* or *F. prausnitzii* (BMDM: bacteria = 1: 20) for 1 h. **A** Cytokine production in BMDM. ***p < 0.001; ns, not significant; by one-way ANOVA with Dunnett’s test. Experiments were repeated 3 times. **B** Immunostaining of IL-1β (red). Phalloidin (green) was used to outline BMDM. **C** Immunostaining of phospho P65 (pP65, red). Phalloidin (green) was used to outline BMDM. **D**–**E** Whole-cell lysis of BMDM treated with bacteria was subjected to Western blot analysis (40 μg per system). Experiments were repeated 3 times. **D** Expression of inflammasome markers in BMDM. **E** The level of inflammasome executors in BMDM. **F** Immunostaining of NLRP3 (red) and caspase-8 (green) to reveal the assembling of inflammasomes. **G** Immunostaining of GSDMD (red). Phalloidin (green) was used to outline BMDM. **H** Cell death was assessed with PI staining. **P < 0.01, ***P < 0.001; by one-way ANOVA with Dunnett’s test. Experiments were repeated 3 times

Continuous interaction between gut microbiota and the host immune system has a vital impact on the systemic inflammatory status of the host. Macrophage is one of the most prominent cytokine producers in the host, whose cytokine secretion could be triggered by the imbalanced intestinal microbiota [51, 52]. Accumulative evidence has demonstrated the hyper-activation of macrophages in the perivascular lesion of CADASIL [9]. We therefore hypothesized that the patient-enriched bacteria modified macrophage activities in the host with CADASIL. *Fusobacterium* has been recognized as an opportunistic pathogen, and *F. varium* has been associated with ulcerative colitis [53]. In this study, *F. varium* was associated with CADASIL (Fig. 1B) and a higher level of pro-inflammatory cytokines (Fig. 5A–C). As such, we next investigate the pro-inflammatory role of *F. varium* on macrophages. *F. prausnitzii* is recognized as an anti-inflammatory next-generation probiotic and therefore was used as a control. Bone marrow-derived macrophages (BMDM) were treated with *F. varium* or *F. prausnitzii* (BMDM: bacteria = 1: 20) for 1 h and were assessed for production of IL-1β, IL-6, and TNF-α by flow cytometry. We found that *F. varium* treatment significantly increased the expression of IL-1β, but not IL-6 and TNF-α (Fig. 6A). The level of IL-1β was further confirmed by immunostaining (Fig. 6B). Those observations are in line with the serum cytokine data of CADASIL patients, where the increase of IL-1β was more significant than IL-6 and TNF-α (Fig. 4A).

Upregulated expression of IL-1β indicated the activation of NF-κB signaling. As revealed by immunostaining, we found that the *F. varium* treated BMDM displayed translocation of phospho-P65 (pP65) into the nucleus, which demonstrated that *F. varium* provoked NF-κB signaling in macrophages (Fig. 6C). NF-κB is the key transcriptional factor that mediates the activation of inflammasome components including NLRP3. Indeed, we observed a higher expression level of NLRP3 in addition to pro-IL-1β (Fig. 6D). To investigate whether *F. varium* activates inflammasomes in macrophages, we examined the level of cleaved gasdermin D (GSDMD), a downstream event of inflammasome activation and the executioner of pyroptosis. The level of GSDMD cleavage was significantly elevated in *F. varium* treated BMDM (Fig. 6D). Furthermore, GSDMD localized at the cell membrane of *F. varium*-treated macrophage (Fig. 6G), and the cell underwent a lytic form of cell death, detected by the uptake of the cell membrane impermeable propidium iodide (Fig. 6H). These observations demonstrated that *F. varium* triggered NF-κB signaling, inflammasome activation, and pyroptosis in macrophages. To further explore which inflammasome was activated in *F. varium*-treated BMDM, we examined the cleavage of caspase-1, caspase-11, and caspase-8. As revealed by Western blot (Fig. 6E), *F. varium* elicited cleavage of caspase-8, but not caspase-1 or caspase-11 in BMDM. Furthermore, co-localization of caspase-8 and NLRP3 was observed by immunostaining (Fig. 6F). These data demonstrated that *F. varium* triggers the assembly of caspase-1-independent noncanonical NLRP3 inflammasomes that recruit caspase-8, an inflammasome type observed previously in LPS + ATP-treated microglia [54].

### *F. varium* infection aggravated systemic inflammation and behavior disorder in mouse model

**Fig. 7:**
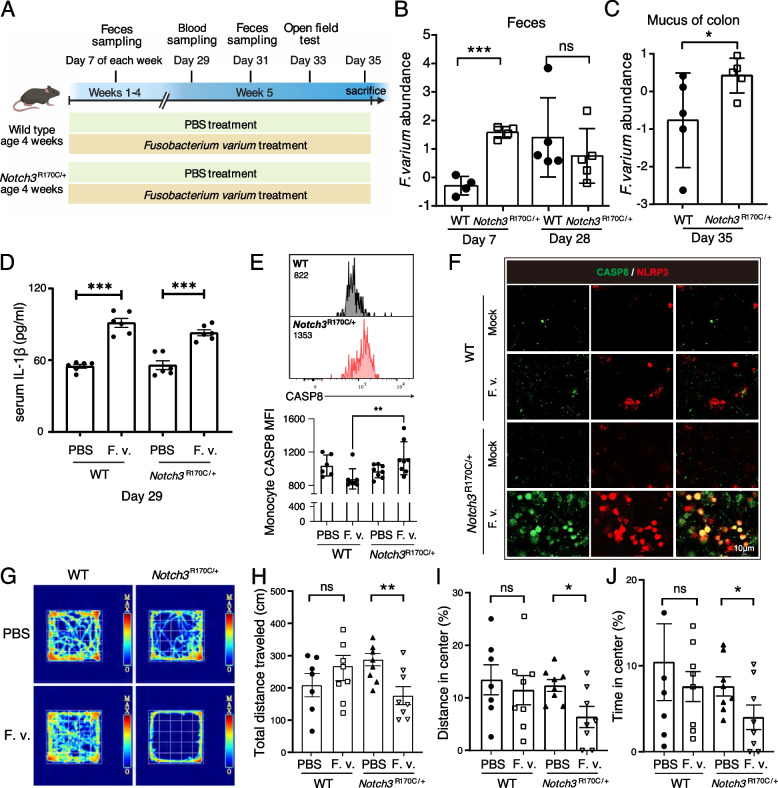
*F. varium* provokes CADASIL-like behaviors and increases systemic inflammation in the mouse model. **A** The mice experimental design. A 4-week-old WT and *Notch3*^R170C/+^ mice received *F. varium* and PBS administration, respectively (*n* = 8 for each group). Feces samples were collected once every week. In the fifth week, blood samples were collected, and an open-field test was performed, and at the last day of this week, all mice were sacrificed, and blood and colon samples were collected. **B**–**C** The abundance of *F. varium*, determined by qPCR, was normalized to pan-bacterial primers targeting the 16S rRNA gene (UNI 16S) in bacterial DNA extracted from feces (**B**) and mucus from colon (**C**) of WT and *Notch3*^R170C/+^ mice. *N* = 5 mice per group. *p*-values were determined by an unpaired Student’s *t*-test. Data are from two independent experiments and represented as the mean ± SD. **D** The comparison of serum IL-1β levels in WT and *Notch3*^R170C/+^ mice after 4 weeks administration of *F. varium* compared to PBS (*n* = 6 for each group). **E** Peripheral blood of *F. varium*-treated mice was subjected to flow cytometric analysis. Monocytes were identified as CD45^+^CD11b^+^F4/80^+^ cells. The mean fluorescent intensity (MFI) of caspase-8 was analyzed. *p*-values were determined by one-way ANOVA with Dunnett’s test. **F** BMDM were isolated and induced from WT and *Notch3*^R170C/+^ mice (age = 8–12 weeks). BMDM were treated with *F. varium* (BMDM: bacteria = 1: 20) for 1 h and subjected to immunostaining of caspase-8, IL-1β, and NLRP3. Experiments were repeated 3 times. **G** Representative figures of the open-field test. The figures were generated with the video tracking system. **H**–**J** The total distance traveled (**H**), the proportion of distance and time in the center (**I**–**J**) in four groups of mice. *p*-values were determined to compare *F*. *varium* and PBS treatment in WT and *Notch3*^R170C/+^ mice separately using an unpaired Student’s *t*-test. *p-value < 0.05; **p-value < 0.01; ***p-value < 0.001; ns, not significant

To further assess whether *F. varium* participates in the pathogenesis of CADASIL at a very early stage before the disease onset, a *Notch3*^R170C/+^ transgenic mouse model was applied. This mouse disease model phenocopies the arteriopathy and the histopathologic as well as clinical features of CADASIL patients and offers opportunities to investigate the roles of bacterial candidates in the pathology of this disease [55]. A 4-week-old *Notch3*^R170C/+^ mice and age- and sex-matched C57BL/6 WT littermates born from the same mothers were gavaged with 10^8^ CFU of *F. varium* every 2 days for 5 consecutive weeks (Fig. 7A). Mice were sacrificed after 5 weeks of treatment and collect their blood and colon mucus samples. Interestingly, compared to wild-type mice, *Notch3*^R170C/+^ mice were more susceptible to the *F. varium* infection. After 1 week of *F. varium* gavage, we were able to detect *F. varium* in the feces of *Notch3*^R170C/+^ mice. In contrast, we did not detect *F. varium* in the feces of WT mice until week 4 (Fig. 7B). At the endpoint of the experiment, we quantified *F. varium* in the colon mucus and observed a higher load of *F. varium* in *Notch3*^R170C/+^ mice compared to WT mice (Fig. 7C).

Next, we examined the systemic inflammation level of the treated mice. Since the abundance of *F. varium* was positively correlated with the level of IL-1β in the human cohort and patient-derived *F. varium* induced the increase of IL-1β in BMDM, we would like to explore whether IL-1β was notably increased after the administration of *F. varium* in mice. As expected, the serum IL-1β was significantly enhanced in the mice with *F. varium* treatment, compared to the PBS-treated ones (Fig. 7D). Notably, *F. varium* gavaged *Notch3*^R170C/+^ mice displayed increased expression of caspase-8 in circulating monocytes (CD45^+^CD11b^+^F4/80^+^ cells in peripheral blood), compared to *F. varium* gavaged WT mice (Fig. 7E)*. Notch3*^R170C/+^ BMDM treated with *F. varium* (BMDM: bacteria = 1:20) had more robust co-localization of NLRP3 and caspase-8 compared to WT BMDM (Fig. 7F). These observations demonstrated *F. varium* promoted inflammation in mice, and the macrophage lineage cells of *Notch3*^R170C/+^ were more susceptible to the activation of the noncanonical NLRP3-caspase-8 inflammasome by *F. varium*.

Furthermore, we assessed the impact of *F. varium* infection on mice behavior. The open-field test showed that *Notch3*^R170C/+^ mice gavaged with *F. varium* were more likely to move along the edges of the open arena and stay in the corner (Fig. 7G). The total distance traveled after *F. varium* treatment was significantly reduced in *Notch3*^R170C/+^ mice (Fig. 7H); meanwhile, the distance and time in the center were also notably decreased in *F. varium* gavaged group compared to the PBS gavaged *Notch3*^R170C/+^ group (Fig. 7I–J). This strongly indicated that *F. varium*-infected mutant mice suffered from an anxiety and behavior disorder, and interestingly, *F. varium* gavage did not bring the same impact on WT mice. Taken together, our data demonstrated that *F. varium* elicited elevated anxiety and impaired behavior in *Notch3*^R170C/+^ mice, and WT mice were resistant to this detrimental impact from *F. varium*.

## Discussion

Although genetic mutations in *NOTCH3* are prerequisites for CADASIL, the environmental factor may have played an unneglectable role in the onset age and severity of this disease. In this study, association and causation studies were conducted. To the best of our knowledge, this is the first effort to investigate the involvement of human gut microbiome in CADASIL in an integrated-omics framework and mouse disease model.

We found out that the alpha diversity and beta diversity of gut microbiota did not exhibit discrepancy between patient and control groups, which was consistent with the conclusion by J. Matsuura *et al*. in 2019 [34]. Further analysis indicates that *Eubacterium eligens* and *Roseburia faecis* were depleted in CADASIL patients (Fig. 1B). *E. eligens* and *R. faecis* are generally recognized as potential probiotics, produce short-chain fatty acids such as butyrate from carbohydrates, and play a role in promoting intestinal health in the host [56]. *Roseburia* has been proven to ameliorate alcohol-related liver diseases [57] and atherogenesis [58] in murine models, while *E. eligens* strongly stimulates the production of the anti-inflammatory cytokine IL-10 in cell-based assays [59]. The depletion of *E. eligens* and *R. faecis* may have been involved in the modulation of local or systemic inflammation, further participation in the progression of CADASIL. Furthermore, the depletion of *R. faecis* contributes to a lower level of four biosynthesis pathways of coenzyme A, thiamine, and flavin in patients (Fig. 1). Thiamine (vitamin B1) is a cofactor for pyruvate dehydrogenase and α-ketoglutarate dehydrogenase, which are both involved in the tricarboxylic acid (TCA) cycle, glutamate, and GABA metabolism. Deficiency of vitamin B1 leads to neuropsychiatric disorders, including Wernicke-Korsakoff syndrome (Wernicke’s encephalopathy and Korsakoff’s psychosis) which manifest as psychosis, delirium, and memory loss [60, 61]. In addition to the defect of thiamin biosynthesis pathways, we have also observed a decreased level of thiamine and allithiamine in patients (Fig. 2), suggesting the deficiency of vitamin B1 might involve in the pathogenesis or severity of the neuropsychiatric disorders present in CADASIL patients, and the depletion of *R. faecis* may have contributed to the lower level of vitamin B1 in patients. As such, the hypothesis that supplementation of vitamin B1 or *R. faecis* might alleviate the neuropsychiatric symptom in CADASIL would be interesting to validate.

The imbalance of Glu/GABA was highly associated with various symptoms of neurological diseases. For example, the ratio of Glu/GABA was significantly increased in the supplementary motor area in patients with ALS [62]. Similarly, the level of serum GABA was decreased in patients with bipolar depression which was linked to the microbiota-gut-brain axis [63]. In a mouse model, it had been confirmed that gut microbiome could modulate the glutamate-glutamine-GABA in schizophrenia [64]. In addition, multiple diseases are associated with an altered GABAergic profile, including depression [65], ASD [66], and cerebral amyloid angiopathy [67]. Our observation of the imbalanced Glu/GABA ratio in CADASIL is in line with these studies. Indeed, in Fig. 1D, the metagenomic sequencing data showed the increased abundance of L-glutamate degradation V pathway in patients; however, the patients also have an increased abundance of 4-aminobutanoate degradation V and GABA shunt pathways which generate glutamate as an intermediate product; as a result, this may have the potential to increase the level of glutamate. Moreover, these two pathways were much more abundant than the L-glutamate degradation V pathway (shown in Fig. 1D). Therefore, it is reasonable to speculate 4-aminobutanoate degradation V, and GABA shunt pathways, *instead of* L-glutamate degradation V, might weigh more in glutamate-GABA metabolism in the gut, and as an overall effect, the level of L-glutamate in serum and feces might be increased. More investigation is needed to evaluate this hypothesis. Furthermore, we showed the increased *Eubacterium siraeum* and *Megasphaera elsdenii* in patient (Fig. 1B) contributed to the elevated abundance of the gene encoding 4-aminobutyrate aminotransferase (EC2.6.1.19), the enzyme that involves in Glu-GABA metabolism and as a result might affect the Glu/GABA ratio (Fig. 3). The genera of *Eubacterium* and *Megasphaera* have been previously reported as GABA-degradation bacteria in human gut microbiota [68], which supports our findings. Notably, although the abundance of *E. siraeum* is < 0.1% in healthy people, their abundance in CADASIL patients is high (1.96%, ranked in the 12th place). The abundance of *M. elsdenii* is undetectable in healthy people but increased to 0.104% in CADASIL patients. Future studies will focus on the mechanisms of *E. siraeum* and *M. elsdenii* in the pathogenesis of CADASIL.

Inflammation has been linked to CADASIL in previous studies [49]. In this study, we revealed the levels of five cytokines increased in CADASIL (Fig. 4); however, the direct inflammation-causing factors remain undetermined. Notably, two CADASIL-enriched bacteria, *Fusobacterium varium* and *Clostridium aldenense*, correlated to the levels of certain cytokines (Fig. 5A), and mono-infection on *Notch3*^R170C/+^ mice verified the role of *F. varium* in promoting inflammation in CADASIL. The relative abundance of *F. varium* was moderate in our CADASIL patients (0.325%), which exceeded the average level (< = 0.11%) of *F. varium* in a southern Chinese population comprising 7009 individuals [69]. Several studies have demonstrated that even at low abundance, microbial pathogens can also orchestrate disease [70]. For example, *Fusobacterium nucleatum* has been well accepted as a key pathogenic factor and microbial biomarker for colorectal cancer (CRC); however, its average relative abundance ranged from 0.010 to 0.073% among different CRC cohorts [71]. This firmly supports the “keystone pathogen” hypothesis that certain minority pathogens are more than capable to stir up trouble in the gut. In previous studies, *F. varium* has been associated with ulcerative colitis [53, 72] and was reported as an emerging pathogen that causes bacteremia [73]. More importantly, the impact of *F. varium* on influencing mood and behavior was revealed in this study for the first time, and whether *F. varium* plays a direct or indirect role in behavior dysfunction in this disease needs further investigation.

Among the elevated cytokines in patients with CADASIL, the increase of IL-1β is the most prominent, which has been appreciated as the leading factor in the NLRP3 inflammasome activation [74], as evidenced by the absence of caspase-8 compromised the consistent accumulation of pro-IL-1β and NLRP3 protein in the macrophages [75, 76]. According to our data, the upregulated IL-1β could be attributed to the activation of noncanonical caspase-8-dependent inflammasome in macrophages by enrichment of *F. varium* in gut microbiota. Microglial caspase-8-dependent inflammasome activation has been found to exacerbate neural inflammation in experimental autoimmune encephalomyelitis [54]. The detrimental impact of microglia activation on CADASIL pathology has been indicated recently [49]. Since *F. varium* induces assembly of caspase-8 dependent inflammasome in macrophages, we reason that the bacteria provoke a similar pro-inflammatory process in microglia, which are brain resident macrophages, and promote neural inflammatory responses in CADASIL. Gut microbiota-brain communication via the immune system is important in modulating host behaviors including anxiety and depression [77].

Gut microbiota is implicated as the second genome of human and has been demonstrated to participate in the pathogenesis of various diseases, including ALS and ASD in which patients carry genetic mutation variants [22, 24]. In turn, the primary genome and its genetic variation of people potentially influence the gut microbiota via a combination of environmental and host factors. In multiple genome-wide association analyses including Dutch Microbiome Project and the MiBioGen consortium [78, 79], people have discovered genetic loci associated with specific bacteria and microbiome composition. Elinav and Segal's groups have reported that the spontaneous colonization of germ-free *Sod1-Tg* mice and their wild-type littermates in the same facility led to dysbiosis of the gut microbiota [22], which indicated the host mutation variant might play an initiative and fundamental role in the establishment of the gut microbiota configuration. Our observations that gut pathobiont *F. varium* was prone to colonize in *Notch3* mutant mice rather than in wild-type mice, endorsed the roles of host genetics in shaping gut microbiota composition. Comprehensive investigation in phenotype discrimination of littermates with wild-type, heterozygous, and homozygous genotypes in *Notch3* mutation animal models is requisite for dissecting functions contributed by host genetics, gut microbiota, or other environmental factors.

The diagnostics for CADASIL have currently relied on expensive genetic tests via whole exome-sequencing approach, which unfortunately has not been widely implemented in most hospitals in China. As such, only a small portion of CADASIL patients are diagnosed and identified, which impeded us to recruit more patients for this study within a limited time. Although the sample size in this study is relatively small, thanks to the powerful integrated-omics analysis, we were able to demonstrate association and provided several hypotheses on causal linkage in the brain-gut-microbe axis. Notably, the hypothetical role of *F. varium* in CADASIL derived from the integrated-omics analysis was experientially validated with cell models and *Notch3* mouse model. Further studies are required to investigate whether disease symptoms can be ameliorated by the modulation of gut microbiota.

## Conclusions

Our results demonstrate that the abundance of several species, correlated functional pathways, serum inflammatory cytokines, and neurotransmitters were altered in CADASIL patients. We establish association links between gut microbiota and the brain in CADASIL (Fig. S10). We also validate in cell and mouse models that *F. varium* promotes systemic inflammation potentially via induction of caspase-8-dependent noncanonical inflammasome activation in macrophages.

### Supplementary Information

**Additional file 1: Fig. S1. **Flow chart and enrolled participants in the current study.

**Additional file 2: Fig. S2. **The relative abundance of significantly different genera in fecal metagenomic samples. The significance levels were calculated by the Wilcoxon rank-sum test. *, *p* value<0.05. Genera names were colored according to the direction of enrichment in the case (orange) or the control (blue) group, respectively. Case, *n*=24; Control, *n*=28.

**Additional file 3: Fig. S3. **Species correlation and co-occurrence network between patients and healthy controls. (**A-B**) The plots display the correlation matrix of significantly differential 10 species in the case group and 9 species in the control group. The abundance of Megasphaera elsdenii was all zero in 28 metagenomic samples from healthy controls, so it showed the correlation of 9 species in the control group. The correlation coefficients were labeled on the correlogram, and red color indicates the positive correlation, while blue indicates the negative correlation. Case, *n*=24; Control, *n*=28. (**C-D**) Species co-occurrence network between patients (**C**) and healthy controls (**D**) based on the Spearman correlation algorithms. Only the top 30 species in relative abundance were shown. Each node presents a bacterial species. The node size indicates the relative abundance of each species per group, and the density of the dashed line represents the Spearman coefficient. Red links stand for a positive correlation between nodes, and blue links stand for a negative correlation, with Spearman's rank correlation coefficient >0.4 and <−0.4, respectively.

**Additional file 4: Fig. S4. **The top 4 significantly decreased microbial pathways in CADASIL patients compared to healthy controls, and the abundance of their contributed species within case and control groups, respectively. The average relative abundance was shown on the right of the stacked bar plots. Species and “unclassified” stratifications are linearly (proportionally) scaled within the total bar height. Case, *N*=24; Control, *N*=28.

**Additional file 5: Fig. S5. **Relative abundance of the top 5 contributing species in the control group to the four pathways (PWY-4242, COA-PWY, PWY-7357, and PWY-6168) and their comparison with that in the case group. Significance was tested by the Wilcoxon rank-sum test. *, *p* value<0.05; ns, not significant.

**Additional file 6: Fig. S6. **Boxplot shows the content of serum GABA in two groups of samples identified by untargeted serum metabolome, and the content of fecal GABA identified by untargeted fecal metabolome.

**Additional file 7: Fig. S7. **Stacked bar plot shows the relative abundance of contributing species to EC2.6.1.19 within a single sample in the case** (A) **and the control** (B) **groups, respectively. The average relative abundance within the group was shown on the right. The sample label indicates the samples come from which group.

**Additional file 8: Fig. S8. **The comparison of VFs in two groups of metagenomic samples.** (A) **The CPM of total VFs in fecal samples from CADASIL patients and controls. **(B)** Significantly enriched VFs in the case group that belong to the T6SS category were shown. Case, *n*=24; Control, *n*=28.

**Additional file 9: Fig. S9. **The significantly decreased virulence factors (VFs) in relative abundance in patients. **(A) **Heat map of the abundance of 35 VFs that enriched in the control group. VFs belonging to the same category were listed together, and these VFs were divided into seven categories. **(B) **Heat map of the Spearman's correlation between the enriched VFs in the control group and six serum inflammatory cytokines.

**Additional file 10: Fig. S10. **The potential brain-gut-microbe axis in CADASIL with NOTCH3 mutation. Gut microbiota may involve the potential pathogenesis of CADASIL through three pathways of communication, including coenzyme A and B vitamins, virulence factors associated with inflammation, and Glu/GABA metabolism.

**Additional file 11: Table S1. **The primers were used to assess the *F. varium* abundance in feces and mucus of the colon from WT and Notch3^R170C/+^ mice.

**Additional file 12: Table S2. **Human study participant details.

**Additional file 13: Table S3. **The metagenomic sequencing data of 52 fecal samples.

**Additional file 14: Table S4. **The species diversity calculated by the Shannon index in fecal metagenomic samples.

**Additional file 15: Table S5. **The significantly different species in relative abundance between CADASIL patients (Case) and healthy family members (Control).

**Additional file 16: Table S6. **The significantly different functional pathways in relative abundance between CADASIL patients and controls.

**Additional file 17: Table S7. **The significantly different content of fecal metabolites between CADASIL patients and healthy controls.

**Additional file 18: Table S8. **KEGG enrichment analysis of differential fecal metabolites.

**Additional file 19: Table S9. **The statistical result of differential serum metabolites by untargeted metabolomic analysis.

**Additional file 20: Table S10. **KEGG enriched pathways for differential serum metabolites between patients and controls.

**Additional file 21: Table S11. **The statistical result of serum neurotransmitters of CADASIL patients (Case) compared to healthy family members (Control).

**Additional file 22: Table S12. **The content of serum glutamic acid and gamma-aminobutyric acid and their ratio in CADASIL patients and controls.

**Additional file 23: Table S13. **The serum cytokines in 12 CADASIL patients (Case) and 10 healthy family members (Control).

**Additional file 24: Table S14. **Differentiated abundant VFs in CADASIL patients compared to controls.

**Additional file 25: Table S15. **Significantly decreased VFs in CADASIL patients compared to controls.

**Additional file 26: Table S16. **The significant correlation between several differential VFs and inflammatory cytokines.

**Additional file 27: Table S17. **The aligning result (identity>=30%) of the genome of identified species in our study to the sequencing of VFG002555 (wcbL) from VFDB.

## Data Availability

All data relevant to the study are included in the article or uploaded as supplementary information. The metagenomic data presented in this study are available in the Genome Sequence Archive (GSA) at the National Genomics Data Center, China National Center for Bioinformation, and the accession number is CRA006871.
