# Supplementary figures and images for: Gut microbes exacerbate systemic inflammation and behavior disorders in neurologic disease CADASIL
Source: Microbiome. 2023 Sep 8;11:202. doi: 10.1186/s40168-023-01638-3 (PMC10486110; doi:10.1186/s40168-023-01638-3)

A

Case

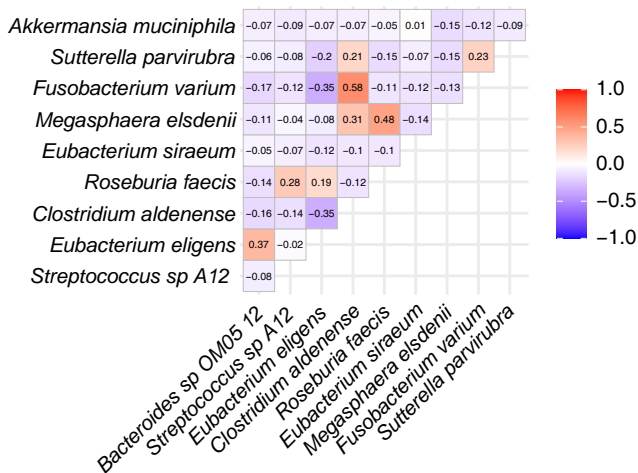

B

Control

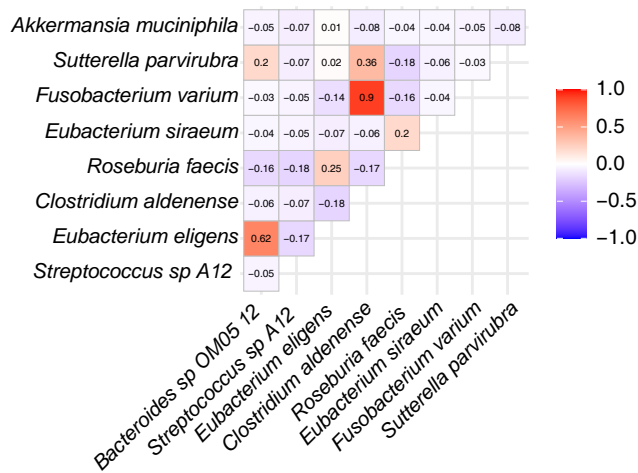

C

Case

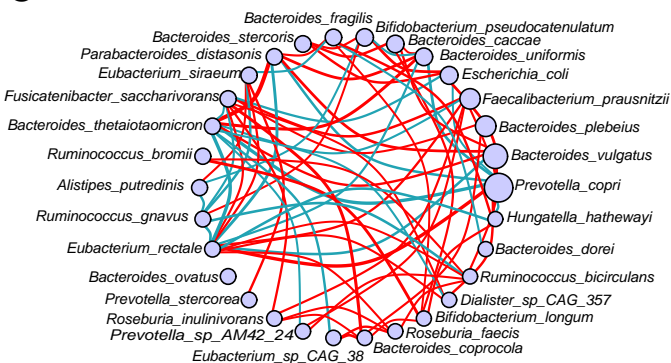

D

Control

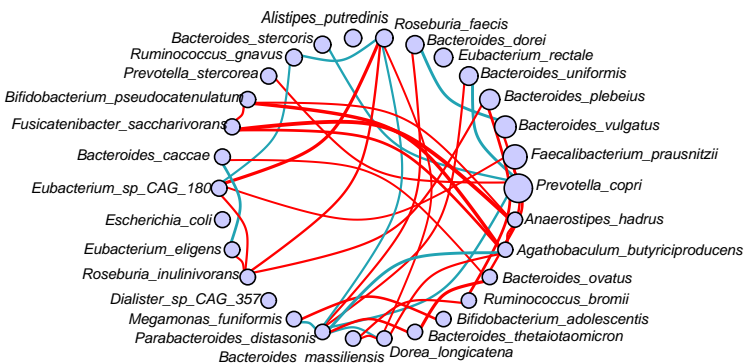

Supplement: Supplementary file 4 — Additional file 3: Fig. S3. Species correlation and co-occurrence network between patients and healthy controls. (A-B) The plots display the correlation matrix of significantly differential 10 species in the case group and 9 species in the control group. The abundance of Megasphaera elsdenii was all zero in 28 metagenomic samples from healthy controls, so it showed the correlation of 9 species in the control group. The correlation coefficients were labeled on the correlogram, and red color indicates the positive correlation, while blue indicates the negative correlation. Case, n=24; Control, n=28. (C-D) Species co-occurrence network between patients (C) and healthy controls (D) based on the Spearman correlation algorithms. Only the top 30 species in relative abundance were shown. Each node presents a bacterial species. The node size indicates the relative abundance of each species per group, and the density of the dashed line represents the Spearman coefficient. Red links stand for a positive correlation between nodes, and blue links stand for a negative correlation, with Spearman's rank correlation coefficient >0.4 and <−0.4, respectively. [file 40168_2023_1638_MOESM3_ESM.pdf]

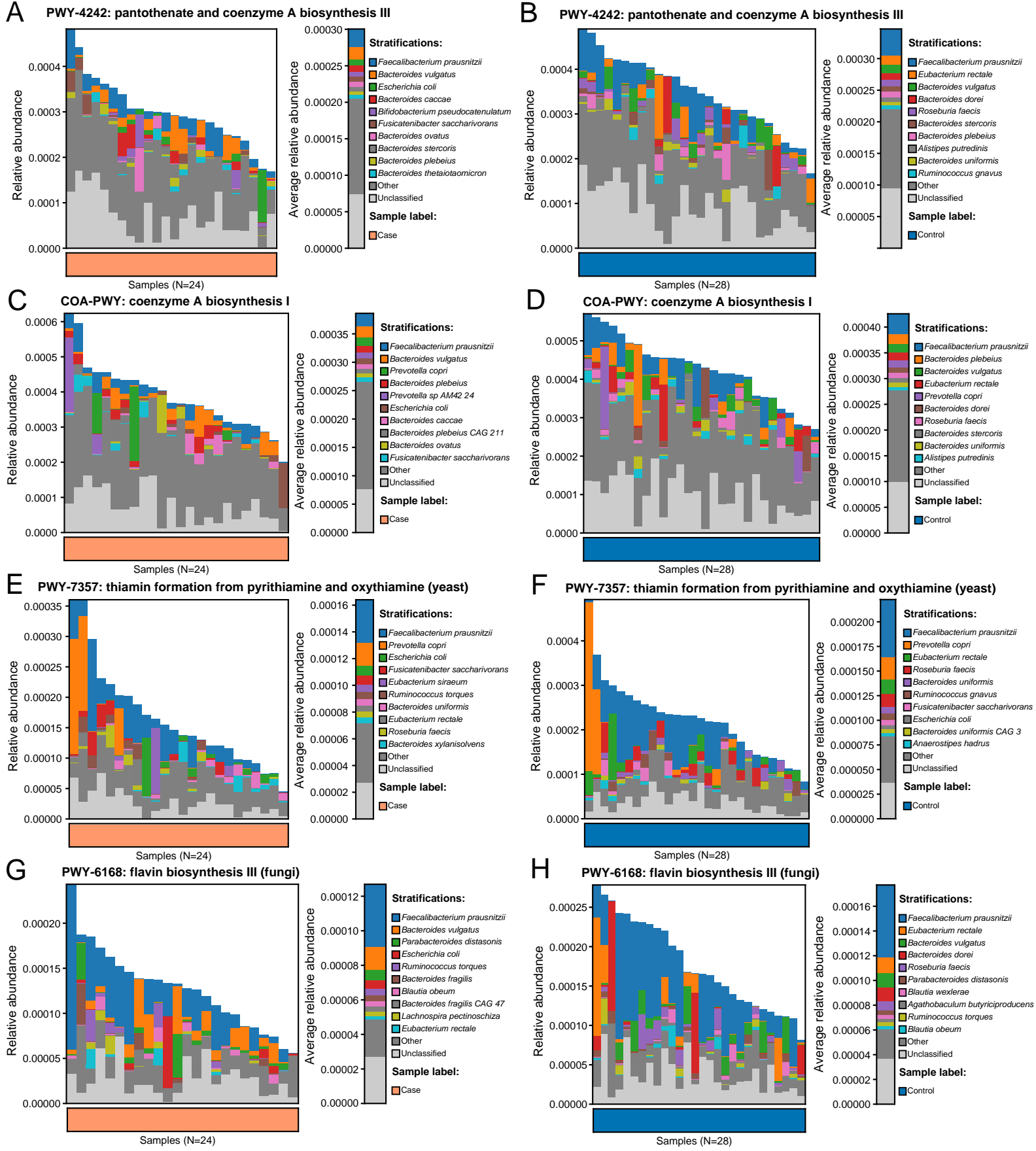

Supplement: Supplementary file 5 — Additional file 4: Fig. S4. The top 4 significantly decreased microbial pathways in CADASIL patients compared to healthy controls, and the abundance of their contributed species within case and control groups, respectively. The average relative abundance was shown on the right of the stacked bar plots. Species and “unclassified” stratifications are linearly (proportionally) scaled within the total bar height. Case, N=24; Control, N=28. [file 40168_2023_1638_MOESM4_ESM.pdf]

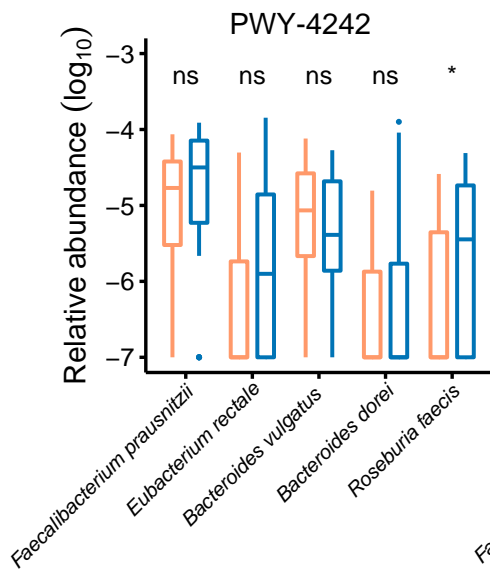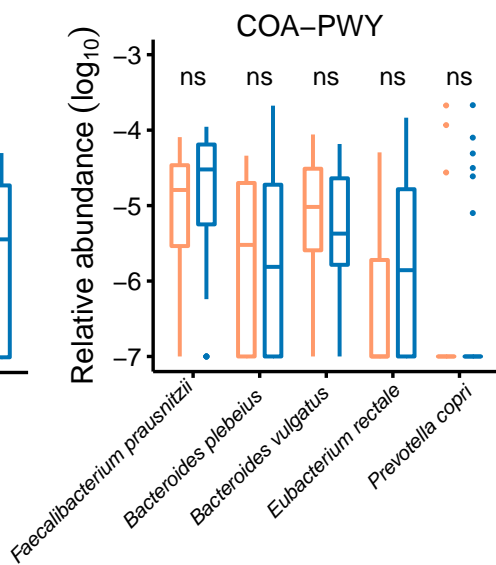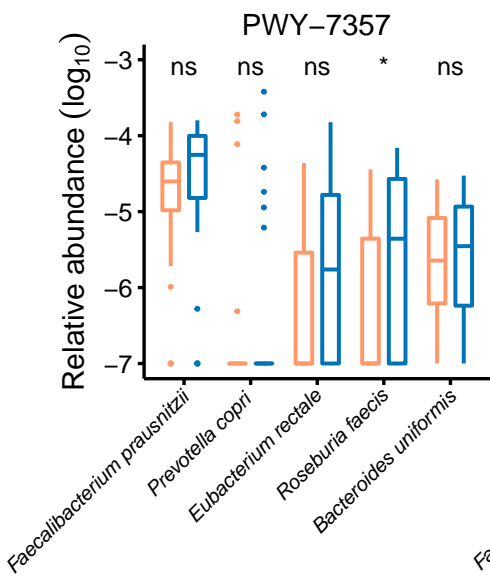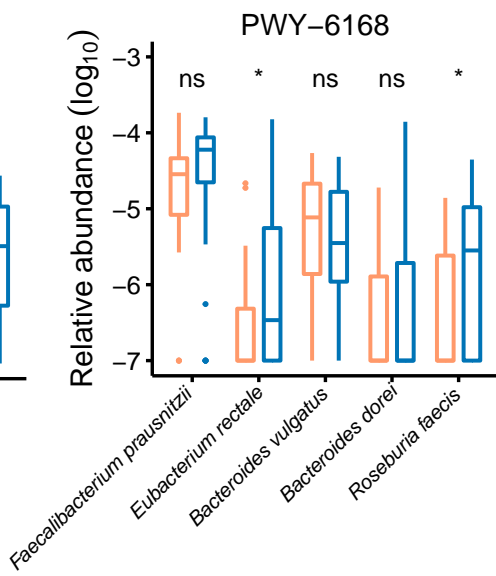

Supplement: Supplementary file 6 — Additional file 5: Fig. S5. Relative abundance of the top 5 contributing species in the control group to the four pathways (PWY-4242, COA-PWY, PWY-7357, and PWY-6168) and their comparison with that in the case group. Significance was tested by the Wilcoxon rank-sum test. *, p value<0.05; ns, not significant. [file 40168_2023_1638_MOESM5_ESM.pdf]

untargeted serum GABA

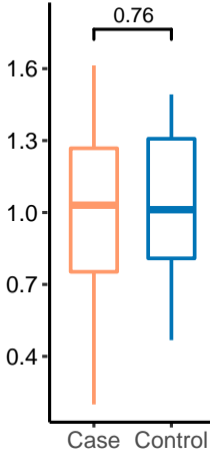

untargeted fecal GABA

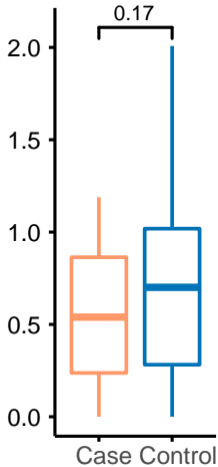

Supplement: Supplementary file 7 — Additional file 6: Fig. S6. Boxplot shows the content of serum GABA in two groups of samples identified by untargeted serum metabolome, and the content of fecal GABA identified by untargeted fecal metabolome. [file 40168_2023_1638_MOESM6_ESM.pdf]

A

2.6.1.19

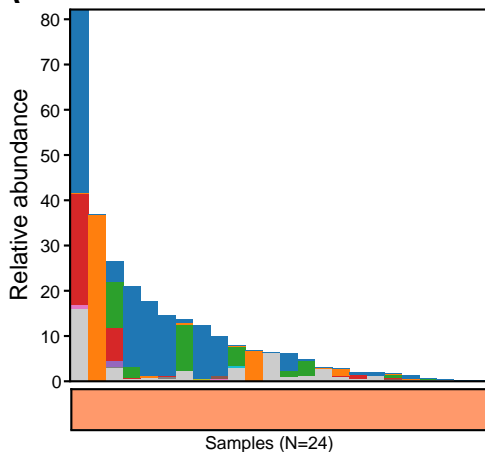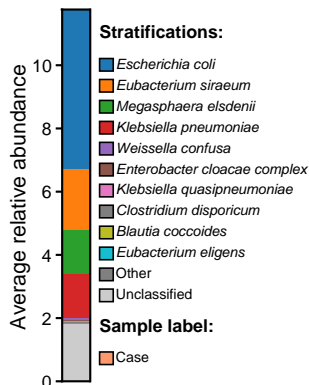

B

2.6.1.19

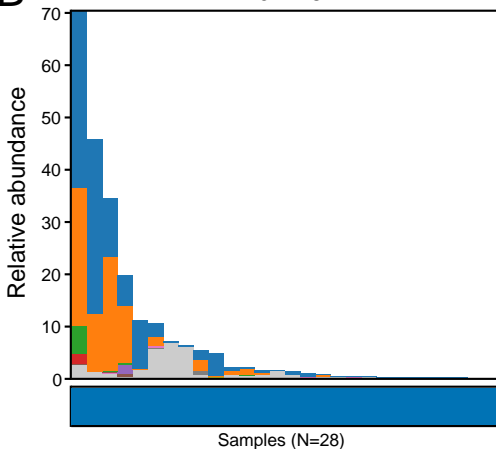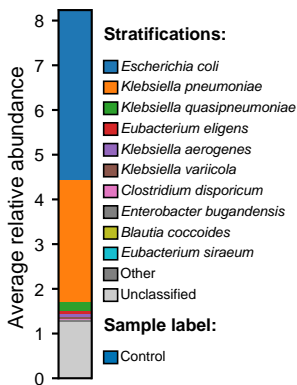

Supplement: Supplementary file 8 — Additional file 7: Fig. S7. Stacked bar plot shows the relative abundance of contributing species to EC2.6.1.19 within a single sample in the case (A) and the control (B) groups, respectively. The average relative abundance within the group was shown on the right. The sample label indicates the samples come from which group. [file 40168_2023_1638_MOESM7_ESM.pdf]

A

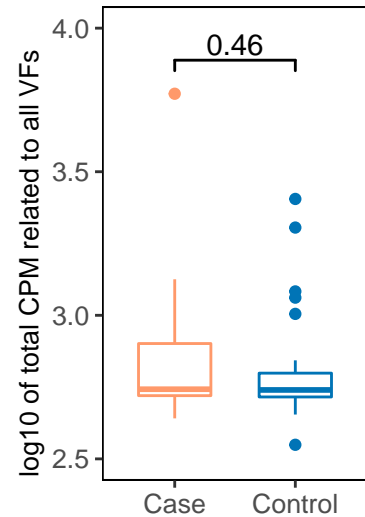

B

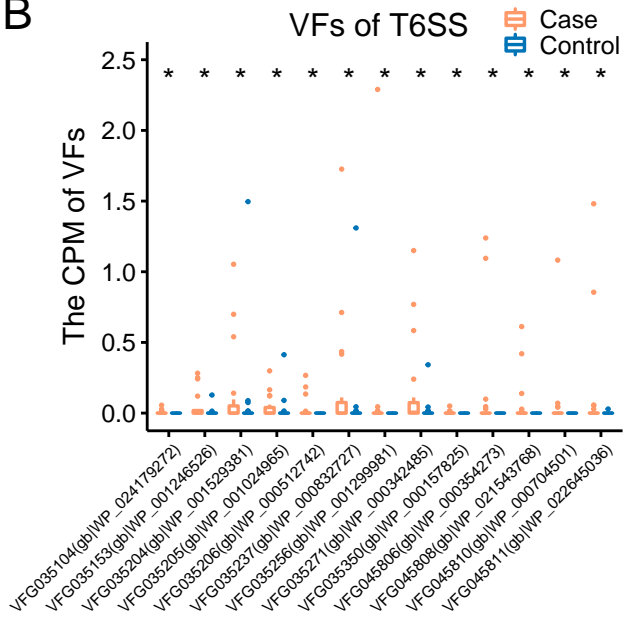

Supplement: Supplementary file 9 — Additional file 8: Fig. S8. The comparison of VFs in two groups of metagenomic samples. (A) The CPM of total VFs in fecal samples from CADASIL patients and controls. (B) Significantly enriched VFs in the case group that belong to the T6SS category were shown. Case, n=24; Control, n=28. [file 40168_2023_1638_MOESM8_ESM.pdf]
