# Supplementary material for: Gut microbes exacerbate systemic inflammation and behavior disorders in neurologic disease CADASIL
Source: Microbiome. 2023 Sep 8;11:202. doi: 10.1186/s40168-023-01638-3 (PMC10486110; doi:10.1186/s40168-023-01638-3)
Supplement: Supplementary file 10 — Additional file 9: Fig. S9. The significantly decreased virulence factors (VFs) in relative abundance in patients. (A) Heat map of the abundance of 35 VFs that enriched in the control group. VFs belonging to the same category were listed together, and these VFs were divided into seven categories. (B) Heat map of the Spearman's correlation between the enriched VFs in the control group and six serum inflammatory cytokines. [file 40168_2023_1638_MOESM9_ESM.pdf]

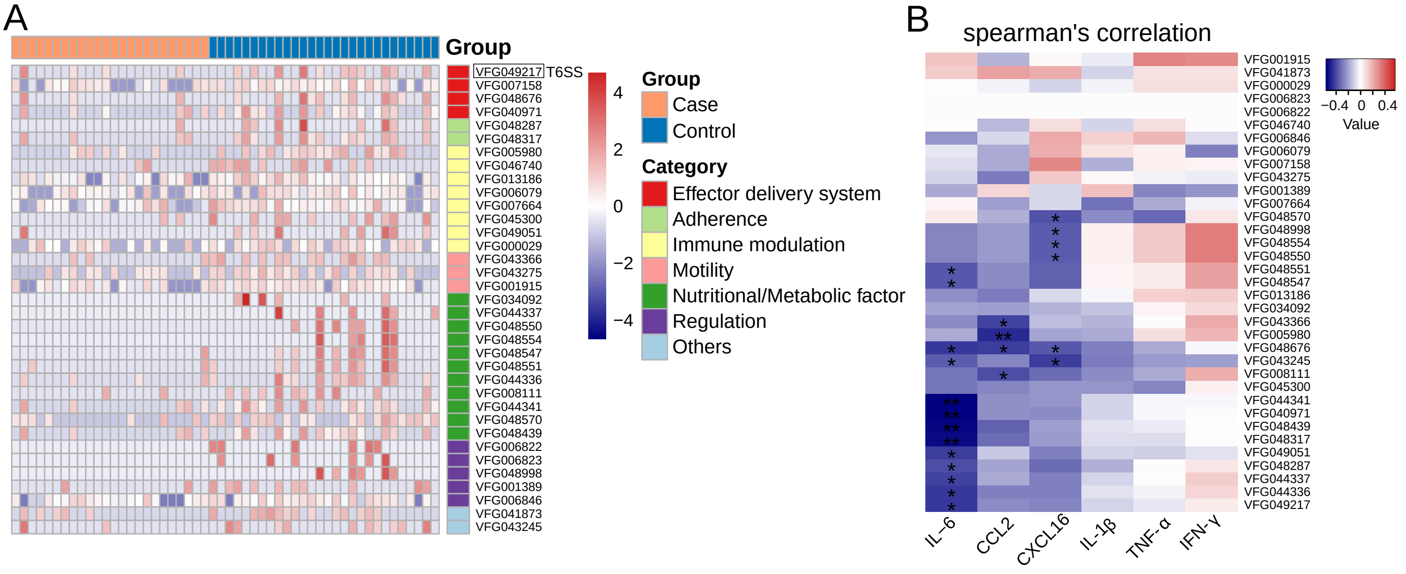
A
Group
VFG049217 T6SS
VFG007158
VFG048676
VFG040971
VFG048287
VFG048317
VFG005980
VFG046740
VFG013186
VFG006079
VFG007664
VFG045300
VFG049051
VFG000029
VFG043366
VFG043275
VFG001915
VFG034092
VFG044337
VFG048550
VFG048554
VFG048547
VFG048551
VFG044336
VFG008111
VFG044341
VFG048570
VFG048439
VFG006822
VFG006823
VFG048998
VFG001389
VFG006846
VFG041873
VFG043245
4
2
0
−2
−4
Group
Case
Control
Category
Effector delivery system
Adherence
Immune modulation
Motility
Nutritional/Metabolic factor
Regulation
Others
B
spearman's correlation
VFG001915
VFG041873
VFG000029
VFG006823
VFG006822
VFG046740
VFG006846
VFG006079
VFG007158
VFG043275
VFG001389
VFG007664
VFG048570
VFG048998
VFG048554
VFG048550
VFG048551
VFG048547
VFG013186
VFG034092
VFG043366
VFG005980
VFG048676
VFG043245
VFG008111
VFG045300
VFG044341
VFG040971
VFG048439
VFG048317
VFG049051
VFG048287
VFG044337
VFG044336
VFG049217
−0.4 0 0.4
Value
IL-6
CCL2
CXCL16
IL-1β
TNF-α
IFN-γ
