# Supplementary material for: Gut microbes exacerbate systemic inflammation and behavior disorders in neurologic disease CADASIL
Source: Microbiome. 2023 Sep 8;11:202. doi: 10.1186/s40168-023-01638-3 (PMC10486110; doi:10.1186/s40168-023-01638-3)
Supplement: Supplementary file 11 — Additional file 10: Fig. S10. The potential brain-gut-microbe axis in CADASIL with NOTCH3 mutation. Gut microbiota may involve the potential pathogenesis of CADASIL through three pathways of communication, including coenzyme A and B vitamins, virulence factors associated with inflammation, and Glu/GABA metabolism. [file 40168_2023_1638_MOESM10_ESM.pdf]

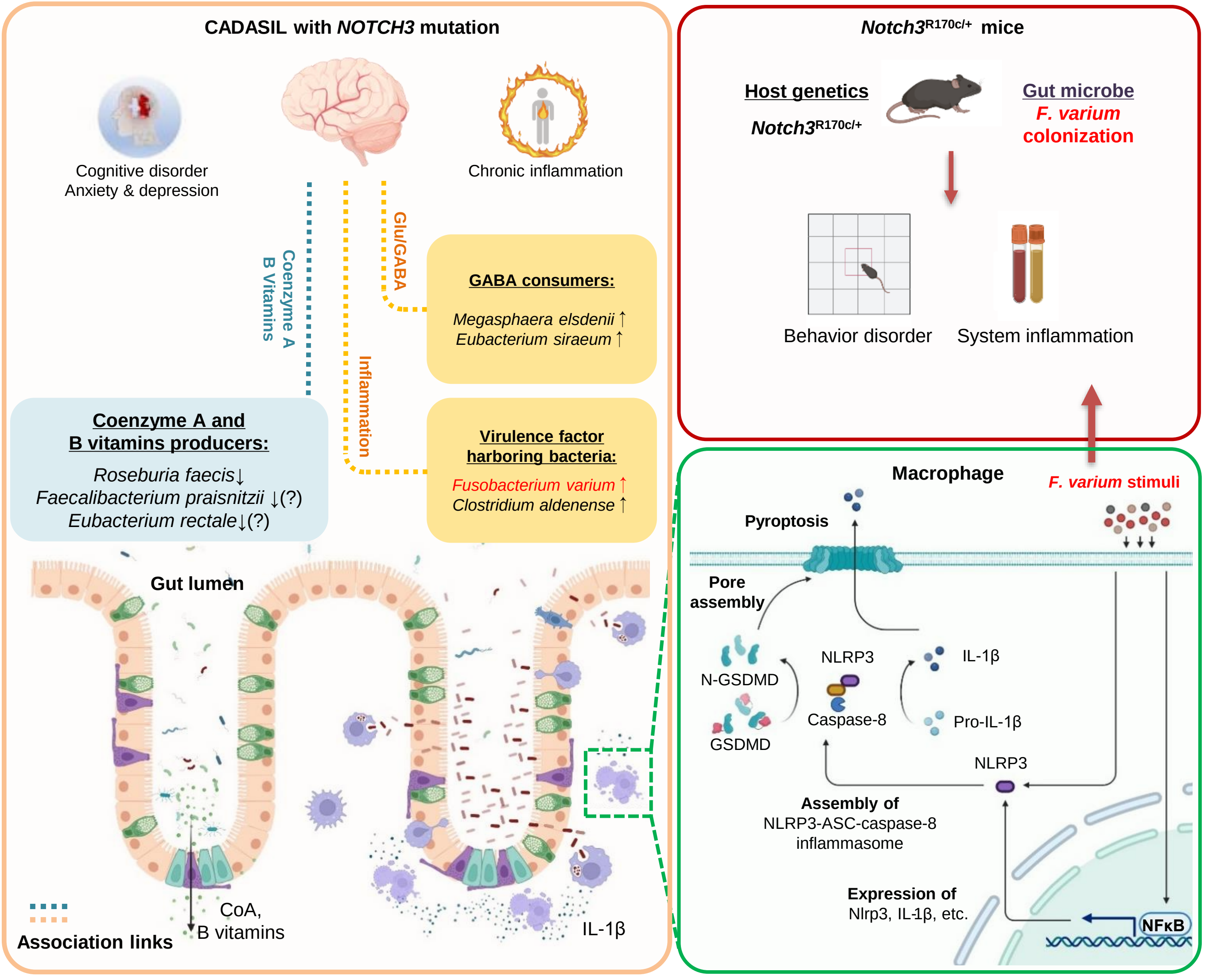

CADASIL with NOTCH3 mutation
Cognitive disorder
Anxiety & depression
Chronic inflammation
Coenzyme A
B Vitamins
Glu/GABA
Inflammation
GABA consumers:
Megasphaera elsdenii ↑
Eubacterium siraeum ↑
Coenzyme A and
B vitamins producers:
Roseburia faecis↓
Faecalibacterium praisnitzii ↓(?)
Eubacterium rectale↓(?)
Virulence factor
harboring bacteria:
Fusobacterium varium ↑
Clostridium aldenense ↑
Gut lumen
CoA,
B vitamins
IL-1β
Association links
Notch3R170c/+ mice
Host genetics
Notch3R170c/+
Gut microbe
F. varium
colonization
Behavior disorder
System inflammation
Macrophage
F. varium stimuli
Pyroptosis
Pore
assembly
N-GSDMD
GSDMD
NLRP3
Caspase-8
IL-1β
Pro-IL-1β
NLRP3
Assembly of
NLRP3-ASC-caspase-8
inflammasome
Expression of
Nlrp3, IL-1β, etc.
NFκB
