# Supplementary material for: Gut microbes exacerbate systemic inflammation and behavior disorders in neurologic disease CADASIL
Source: Microbiome. 2023 Sep 8;11:202. doi: 10.1186/s40168-023-01638-3 (PMC10486110; doi:10.1186/s40168-023-01638-3)
Supplement: Supplementary file 2 — Additional file 1: Fig. S1. Flow chart and enrolled participants in the current study. [file 40168_2023_1638_MOESM1_ESM.pdf]

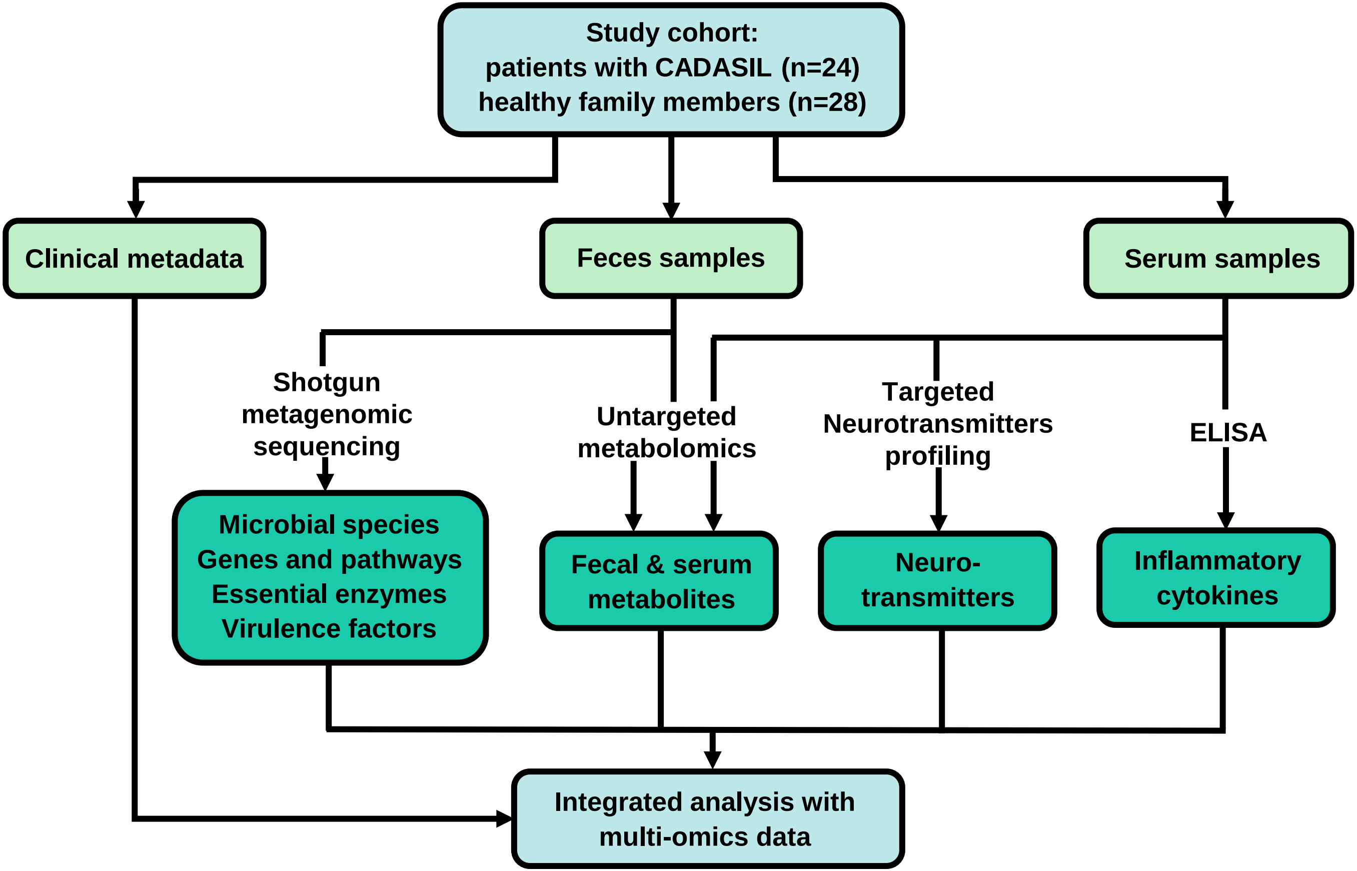
Study cohort:
patients with CADASIL (n=24)
healthy family members (n=28)
Clinical metadata
Feces samples
Serum samples
Shotgun metagenomic sequencing
Untargeted metabolomics
Targeted Neurotransmitters profiling
ELISA
Microbial species
Genes and pathways
Essential enzymes
Virulence factors
Fecal & serum metabolites
Neuro-transmitters
Inflammatory cytokines
Integrated analysis with multi-omics data
