# Supplementary material for: Gut microbes exacerbate systemic inflammation and behavior disorders in neurologic disease CADASIL
Source: Microbiome. 2023 Sep 8;11:202. doi: 10.1186/s40168-023-01638-3 (PMC10486110; doi:10.1186/s40168-023-01638-3)
Supplement: Supplementary file 3 — Additional file 2: Fig. S2. The relative abundance of significantly different genera in fecal metagenomic samples. The significance levels were calculated by the Wilcoxon rank-sum test. *, p value<0.05. Genera names were colored according to the direction of enrichment in the case (orange) or the control (blue) group, respectively. Case, n=24; Control, n=28. [file 40168_2023_1638_MOESM2_ESM.pdf]

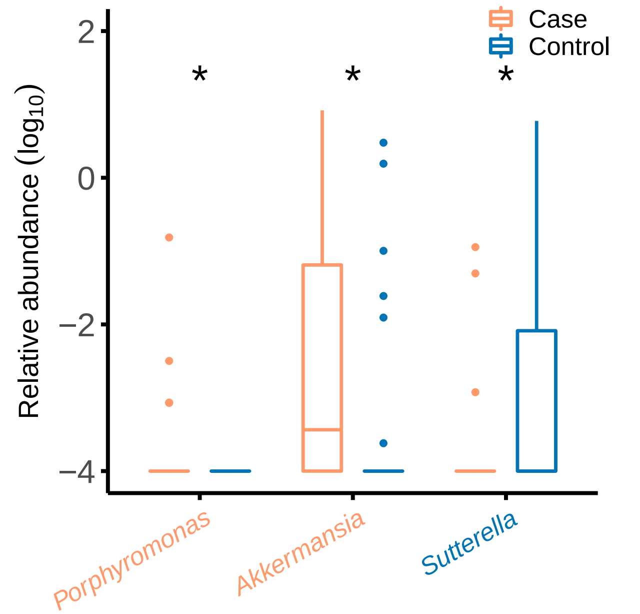

Case
Control
*
*
*
Relative abundance (log10)
2
0
−2
−4
Porphyromonas
Akkermansia
Sutterella
